# Genome mining guided identification of new furo[3,2-*c*]pyridones phaeopyridones A–C from the endophytic fungus *Phaeobotryon negundinis* 12020

**DOI:** 10.1007/s13659-026-00644-w

**Published:** 2026-08-28

**Authors:** Ke Ye, Jie Shi, Huimin Wu, Shujie Wang, Zexu Lin, Jiale Sun, Yuwei Chen, Bohao Wang, Rongxuan Wang, Chenglin Jiang, Yi Jiang, Tom Hsiang, Bin Zhu, Lixin Zhang, Xueting Liu, Guoliang Zhu

**Affiliations:** 1 State Key Laboratory of Bioreactor Engineering, East China University of Science and Technology, Shanghai, 200237 China; 2 Nanjing Technology University, Nanjing, 211816 China; 3 The Key Lab for Research & Development of Actinomycete Resources, Yunnan Institute of Microbiology, Yunnan University, Chenggong District, Kunming, 650500 China; 4 School of Environmental Sciences, University of Guelph, Guelph, ON N1G 2W1 Canada

**Keywords:** Flavipucine alkaloids, Furo[3,2-*c*]pyridones, Endophytic fungi, Feature-based molecular networking

## Abstract

Three pairs of new furo[3,2-*c*]pyridone derivative racemates, named (±)-phaeopyridones A–C (**1**–**3**), together with two new compounds, berkeleyamide E (**4**), and phaeopyranone (**5**), were isolated from the endophytic fungus *Phaeobotryon negundinis* 12020, along with three known compounds, (8*S*,9*S*)-dihydroisoflavipucine (**6**), (8*S*,9*R*)-dihydroisoflavipucine (**7**), and sapinopyridione (**8**), guided by bioinformatics and feature-based molecular networking and fragment-based LC-MS/MS analysis. The chemical structures of these compounds were elucidated by comprehensive spectroscopic analyses and single-crystal X-ray diffraction. Furthermore, a biosynthetic pathway for compounds **1**–**3** was proposed, indicating the unique furo[3,2-*c*]pyridone moiety may be generated by a non-enzymatic intermolecular hemiketal formation reaction. Our research expands the known range of flavipucine alkaloids and highlights the unexplored biosynthetic potential of natural products derived from endophytic fungi.

**Supplementary Information:**

The online version contains supplementary material available at https://doi.org/10.1007/s13659-026-00644-w.

## Introduction

Pyridones are a distinctive class of nitrogen-containing heterocycles characterized by a six-membered aromatic ring bearing both a carbonyl group and a nitrogen atom. They are classified into 2-pyridones and 4-pyridones. Pyridones serve as privileged scaffolds in drug discovery, functioning as hydrogen bond donors or acceptors and bio-isosteres and enhancing drug-like properties [1]. Although tautomerization can occur between hydroxypyridine and its corresponding pyridone [2], the pyridone form generally predominates and exhibits unique chemical properties, as precursors in the synthesis of other important *N*-heterocycles [3]. Over the past decade, pyridones have emerged as a highly significant nitrogen heterocycles among FDA-approved pharmaceuticals [4], with applications spanning multiple therapeutic areas, including anti-infective, respiratory, cardiovascular, oncology, and central nervous system disorders. The increasing number of FDA-approved drugs featuring pyridone scaffolds over the past two decades further underscores their growing importance in medicinal chemistry [2, 5].

Both 2- and 4-pyridones are critical structural motifs found in numerous natural products and they display a wide spectrum of biological activities, including hypoglycemic [6], antimicrobial [7], antitumor [8], neurotrophic [9], anti-inflammatory [10] and insecticidal properties [11]. As an important class of electron-deficient heterocycles [12], 2-pyridones are produced widely by fungi and bacteria [11]. Among fungal-derived 2-pyridone compounds, flavipucine, featuring by a unique spiro-epoxide moiety, was identified as a phytotoxin [13]. Flavipucine and its derivatives subsequently have been isolated from several fungal species [13–18]. Biological evaluations have demonstrated that these derivatives possess notable antibacterial and antifungal properties [14, 15]. However, structural modifications of flavipucine for lead optimization remain limited, and the reported modifications to date have not substantially enhanced antimicrobial activity [16]. Therefore, exploring natural flavipucine analogs with improved biological activities continues to be of considerable interest.

The flavipucine biosynthetic gene cluster (*flv* BGC) has been identified through heterologous expression in *Aspergillus terreus*. This cluster encodes a hybrid polyketide synthase-nonribosomal peptide synthetase (NRPS-PKS FlvA), a hydrolase (FlvD), two transcription factors, two EthD domain-containing proteins, an MFS-type transporter, and a hypothetical protein (FlvB) [17, 18]. FlvA produces triketide-amino acid via reductive release, FlvD catalyzes first ring cyclization, while FlvB is implicated in a C–N bond oxidative cleavage process of the pyrrolinone moiety, leading to the formation of the 2-pyridone scaffold [19].

Advances in bioinformatics and genetic analytical tools have opened new avenues for the discovery of natural products [19]. Analytical methods for biosynthetic gene clusters (BGCs), such as homology grouping, sequence similarity search, and BGCs visualization, are indispensable for their discovery. Tools, such as BiG-SLiCE [20], BiG-SCAPE [21], cblaster [22], and MultiGeneBlast [20, 23], facilitate local BGC analyses, while Clinker [24] and CORASON [21] enable visualization and comparative genomics, highlighting syntenic and evolutionary relationships between BGCs. Moreover, CAGECAT [25] provides a convenient and BLAST-like web server for BGC analysis. These methods are expected to accelerate the functional analysis and annotation of BGCs, leading to a broader understanding of their downstream products and offering abundant opportunities to expand structural diversity and explore pharmacological potential. Molecular Networking (MN) is a powerful strategy for analyzing chemical diversity by correlating MS^2^ data to reveal fragmentation and ionization patterns. It effectively identifies uncharacterized metabolites with bioactivity or novel biosynthetic origins and enables robust comparisons across time courses or genetic variants. Feature-based Molecular Networking (FBMN**)**, introduced in 2020, enhances MN through chromatographic feature alignment, improved spectral matching, and metabolite annotation [26].

In the study, we characterized several flavipucine-related furo[3,2-*c*]pyridone derivatives from the endophytic fungus *Phaeobotrypon negundinis* 12020, by means of a combinatorial genome mining and fragment-based LC-MS/MS analysis. Further phylogenetic analysis and biosynthetic pathway analysis revealed an FAD-dependent monoxgenase, and that non-enzymatic intramolecular hemiketal formation reaction may result in the formation of unique furo[3,2-*c*]pyridone moieties in (±)-phaeopyridones A–C (**1**–**3**).

## Materials and methods

### Fungal strain isolation, identification, genome sequencing, and *in silico* analysis

The endophytic fungal strain *P. negundinis* 12020 was isolated from *Juniperus* sp. in Ottawa, Ontario, Canada. This strain has been deposited in the China General Microbiological Culture Collection Centre (accession no. CGMCC41217) and stored at − 80 °C. This strain was cultured on Potato dextrose agar (PDA, glucose 20 g/L, potato extract 4 g/L, agar 15 g/L). The genomic DNA extraction of *P. negundinis* 12020 was carried out using CTAB (cetyltrimethylammonium bromide) method following a previously reported method [27]. The ITS region was amplified with primers (F: TCCTCCGCTTATTGATATGC, R: GGAAGTAAAAGTCGTAACAAGG), and the resulting sequences were compared to GenBank using BLAST. Related sequences were downloaded and using CLUSTAL W followed by generating a Neighbor-Joining phylogenetic tree in MEGA 7.0 (Fig. S1A and S1B).

The 2ndFind platform (http://biosyn.nih.go.jp/2ndFind/) was used to locate specific gene clusters, followed by BLASTp (https://blast.ncbi.nlm.nih.gov/Blast.cgi) of specific proteins to search for homologues of Orf12 and predict potential gene functions. Biosynthetic gene cluster comparison was conducted using CAGECAT [25]. The sequence alignment of Orf12 and its homologues was performed using CLUSTAL W in MEGA 7.0. All sequences were subjected to phylogenetic analysis by the Neighbor-Joining method to generate a tree which was visualized with iTOL (https://itol.embl.de/).

### Fungus cultivation, extraction, and isolation

*P. negundinis* 12020 cells were cultured on PDA plates at 28 °C for 10 days. The agar plugs were cut into about 0.25 cm^2^ pieces under aseptic conditions, and transferred into ten Erlenmeyer flasks (250 mL), each containing 100 mL of Potato Dextrose Broth (PDB). For preparing the seed cultures, the Erlenmeyer flasks were incubated at 28 °C for 7 days on a rotary shaker at 220 rpm. Solid rice culture medium was used for fermentation. Aaseptic bags, each filled with 80 g of rice and 120 mL distilled H_2_O, were sterilized at 121 °C for 30 min. The 250 bags were subsequently inoculated with 3 mL seed culture each and fermented at 28 °C for 30 days.

The rice-fermented cultures were subjected to extraction using ethyl acetate (EtOAc), and evaporation of the solvent afforded a crude extract (141.8 g). The crude extract was then suspended in distilled water and extracted consecutively partitioned three times with hexane and EtOAc, which were then evaporated to yield the hexane extract (83.2 g) and the EtOAc extract (38.3 g), respectively. The EtOAc extract was fractionated using a Sephadex LH-20 column (MeOH) to afford seven fractions (G1–G7). Fraction G2 (31.9 g) was further fractionated using Sephadex LH-20 column (MeOH) to obtain eight sub-fractions (G2G1–G2G8). G2G4 (21.8 g) was separated using a Fuji reverse phase-C18 silica gel chromatography column with a MeOH-water gradient system (5:95–100:0, *v*/*v*) to yield eight fractions (G2G4S1–G2G4S7). The G2G4S1 fraction was separated by preparative HPLC using a Basic C18 column at 16.0 mL/min with a gradient elution, where over 0–15 min acetonitrile (ACN) was increased from 10% to 99% in H_2_O with 0.1% formic acid (FA), followed by further purification using a Morphing C18T column to obtain **5** (1.4 mg, *t*_R_ = 11.3 min). The G2G4S4 fraction was separated by semi-preparative HPLC with a ZORBAX SB-C18 column with elution using MeOH-H_2_O (32%–68%) at a flow rate 4.0 mL/min to afford compound **4** (2.8 mg, *t*_R_ = 12 min). The LC–MS analysis of these fractions revealed pyridone compounds in fraction G2G4S3 based on characteristic UV absorption and MS spectra. Then fraction G2G4S3 (1.9 g) was subjected to by preparative HPLC using a Basic C18 column at 16.0 mL/min in a gradient to obtain 20 fractions (G2G4S3H1–G2G4S3H20); (over 0–15 min, ACN was increased from 10% to 99% in H_2_O with 0.1% formic acid solution and then the column was eluted with 100% ACN for 5 min). The fraction G2G4S3H13 was purified by semi-preparative HPLC using ZORBAX SB-C18 column at flow rate of 4.0 mL/min using 34% MeOH-H_2_O to obtain compound **1** (10.1 mg, *t*_R_ = 20.9 min). The fraction G2G4S3H15 was purified by semi-preparative HPLC using a Morphing C18T column at flow rate of 4.0 mL/min with 42% MeOH–58% H_2_O (0.1% FA) to obtain compound **2** (4.1 mg, *t*_R_ = 16.0 min). The fraction G2G4S3H16 was purified by semi-preparative HPLC using a Morphing C18T column at 4.0 mL/min with 35% MeOH–65% H_2_O (0.1% FA) to obtain compound **3** (5.1 mg, *t*_R_ = 16.0 min). Then fraction G2G4S4 (8 g) was isolated by preparative HPLC using ACE C18-PFP column at flow rate of 16.0 mL/min in gridient eliton of 35% MeOH–65% H_2_O (0.1% FA) to 99% MeOH–1% H_2_O (0.1% FA) to obtain compounds** 6** (4.2 mg, *t*_R_ = 19.8 min), **7** (4.3 mg, *t*_R_ = 15 min), and **8** (1.1 mg, *t*_R_ = 12 min).

### Crystallographic data and X-Ray structure analysis of compounds 1–3 and 5 

The bulky crystals of compounds **1**–**3** and** 5** were obtained in the solvent MeOH/H_2_O (4:1, *v*/*v*) at 4 °C. A Bruker APEX-IICCD diffractometer with CuK*α* radiation (*λ* = 1.542) was used to collect single-crystal X-ray data of the compounds., the structures were solved and then were refined with the ShelXL refinement package in Olex2. Crystallographic data for the structures of **1**–**3** and **5** have been deposited in the Cambridge Crystallographic Data Centre (www.ccdc.cam.ac.uk, deposition numbers: 2443614, 2443615, 2443616, and 2443617). Crystal Data for (±)-phaeopyridone A (**1**), (±)-phaeopyridone B (**2**), (±)-phaeopyridone C (**3**), and phaeopyranone (**5**) can be found in Table S4.

### Antimicrobial assays

Compounds were evaluated for their antimicrobial activities against *Candida albicans* ATCC10231, methicillin-resistant *Staphylococcus aureus* ATCC 6538, *Escherichia coli* ATCC11775, *Edwardsiella tarda* ATCC15947, *Bacillus cereus* ATCC14579, *Acinetobacter baumannii* ATCC19606, *Enterobacter aerogenes* ATCC13048, and *Mycobacterium tuberculosi*s H37Ra*.* The assays were performed following the previously reported method [28].

## Results and discussion

### Genome mining for flavipucine alkaloids BGCs

**Fig. 1:**
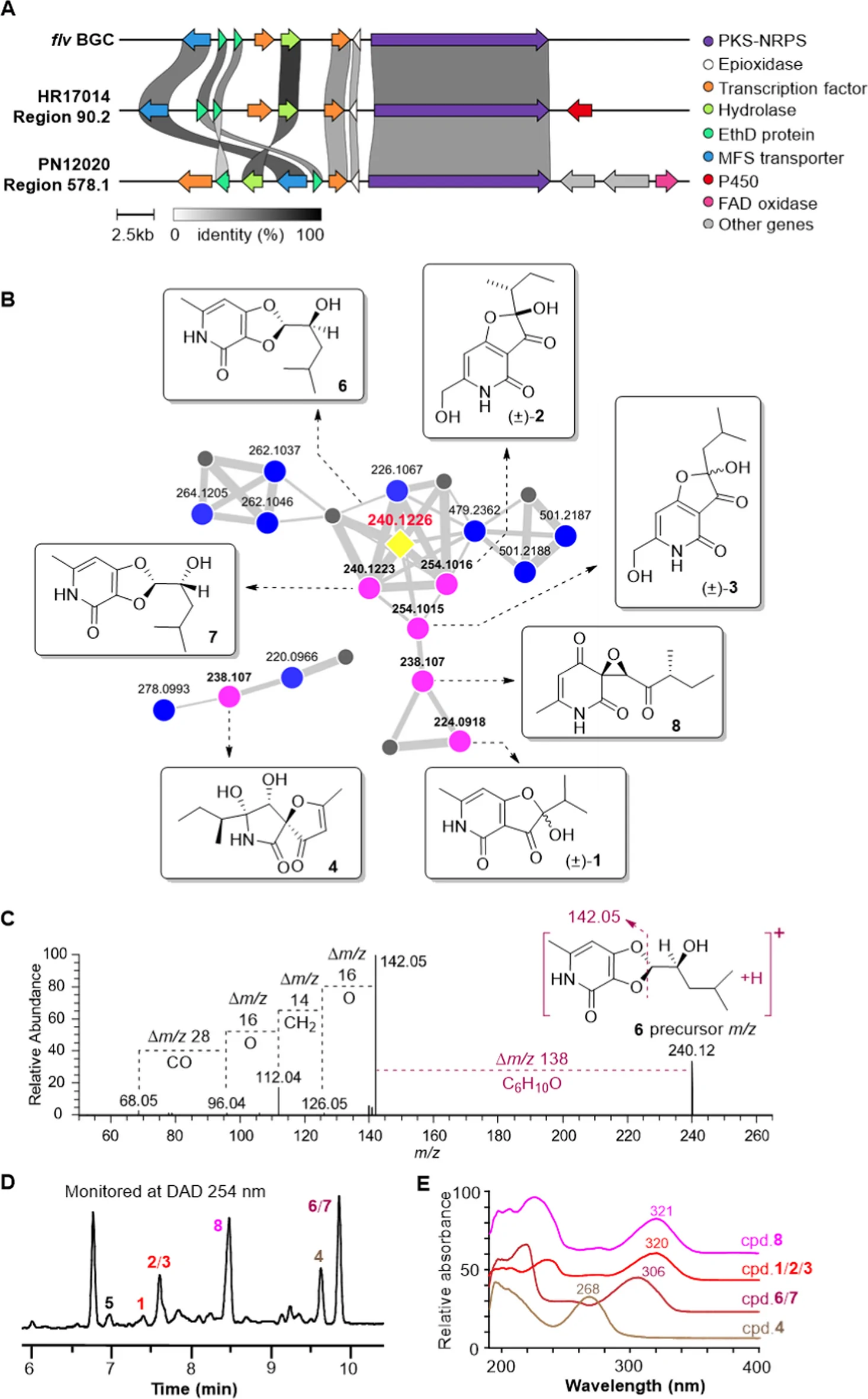
Genome mining and FBMN-guided characterization of new flavipucine alkaloids from *P. negundinis* 12020. **A** The flavipucine alkaloids biosynthetic gene cluster comparison between *A. terreus* NIH2624 (*flv* gene cluster), *H. roseus* 17014, and *P. negundinis* 12020. **B** Clustering of flavipucine analogs. (8*S*,9*S*)-dihydroisoflavipucine (**6**) was used as seed compound (yellow shaded square) in FBMN analysis. **C** MS/MS analysis of known flavipucines. **D** HPLC–UV chromatogram of flavipucines fraction G2G4 examined at 254 nm. **E** The UV spectra of known flavipucine alkaloids

A previously reported *flv* BGC consists of genes encoding a PKS-NRPS (*flvA*), an *α/β* hydrolase (*flvD*), two transcription factors (*flvC* and *flvE*), two EthD-domain-containing proteins (*flvF* and *flvG*), an MFS drug transporter (*flvH*), and a hypothetical protein (*flvB*) [17]. In this biosynthetic pathway, FlvA (PKS-NRPS) catalyzes the biosynthesis of triketide-amino acid via reductive release and FlvB results in the oxidative bond cleavage of pyrrolinone to generate 2-pyridone moiety. Using FlvA and FlvB as probes, 20 fungal strains containing both homologue proteins were identified from an in-house database of over 400 fungal genomes. Functional annotation of approximately 20 kb upstream and downstream regions surrounding FlvAB homologou proteins revealed the presence of a flavipucine biosynthetic gene cluster in two strains from our fungal genome database (Table S1 and S2). A comparative genomic analysis was performed by aligning these newly identified clusters with the *flv* gene cluster from *A. terreus* NIH2624 using Clinker on CAGECAT [25] (Fig. 1A).

The BGCs of *P. negundinis* 12020 and *Hypomyces roseus* 17014 contain key genes involved in flavipucine biosynthesis and exhibit significant similarity and identity to genes of *flv* gene cluster. Notably, the BGC of *P. negundinis* 12020 was found to include *orf12* encoding a potential FAD-dependent monooxygenase, while the *H. roseus* 17014 BGC acetonitrile contain gene *orf10* encoding a potential cytochrome P450 monooxygenase, both of which are absent from the *flv* gene cluster of *A. terreus* NIH2624 (Table S1 and S2). BLASTp analysis of Orf12 from the Swiss-Prot database identified Frs3 as the top hit. Frs3 is a multifunction monooxygenase and responsible for oxidizing 6-*O*-demethylfusarubinaldehyde to generate the hydroxylated substrate [29]. It is plausible that the FAD-dependent monooxygenase encoded by *orf12* catalyzes the oxidative modifications on the pyridone scaffold, potentially playing a crucial role in the biosynthetic pathway. These findings highlight the genomic potential of *P. negundinis* 12020 for production of novel flavipucine alkaloids and generation of structurally modified analogues. Small-scale fermentations of the two strains were conducted using rice solid medium and preliminary HR-MS analysis revealed the production of flavipucine-related products only in extract of *P. negundinis* 12020, but not in *H. roseus* 17014. Consequently, large-scale fermentation of *P. negundinis* 12020 was subsequently undertaken.

### FBMN and Fragment-based LC–MS/MS analysis guided detection of flavipucine alkaloids from *P. negundinis* 12020

The fungal strain *P. negundinis* 12020 was fermented with rice medium and previously reported flavipucine alkaloids (8*S*,9*S*)-dihydroisoflavipucine (**6**) was characterized from the crude extract based on the HR-MS data and UV spectrum [30]. For detection of novel compounds through fermentation using extremely small fermentation quantities, FBMN analysis was conducted using (8*S*,9*S*)-dihydroisoflavipucine (**6**) as the seed compound (Fig. 1B), and the related nodes were found within Clusters I and II (Table S3) consisting of 22 nodes. The HR-ESI-MS data annotation (Table S3), together with further MS/MS analysis comparison to **6**, revealed several potential new compounds that shared fragment ion of *m*/*z* 142.05 (C_6_H_8_NO_3_) or *m*/*z* 126.05 (C_6_H_8_NO_2_) (Fig. 1C and Fig. S3), indicating that they belong to flavipucine alkaloids. In the HPLC chromatogram of subfraction G2G4, these compounds exhibited UV spectra also similar to that of **6** (Fig. 1D and E). Subsequent detailed annotation of the HR-ESI-MS data identified four compounds with previously unreported *m/z* values (224.0918, 254.1015, 254.1016, and 278.0993), which guided the targeted purification of new compounds from subfractions G2G4S1–G2G4S7. As a result, three pairs of new furo[3,2-*c*]pyridone derivative racemates, named (±)-phaeopyridones A–C (**1**–**3**), and a new berkeleyamide (**4**) were obtained, along with several known compounds, (8*S*,9*S*)-dihydroisoflavipucine (**6**) and (8*S*,9*R*)-dihydroisoflavipucine (**7**), and sapinopyridione (**8**) [31]. Through comprehensive spectroscopic analysis and single-crystal X-ray diffraction, the chemical structures of these compounds were elucidated.

### Structure elucidation and antimicrobial activity evaluation

**Fig. 2:**
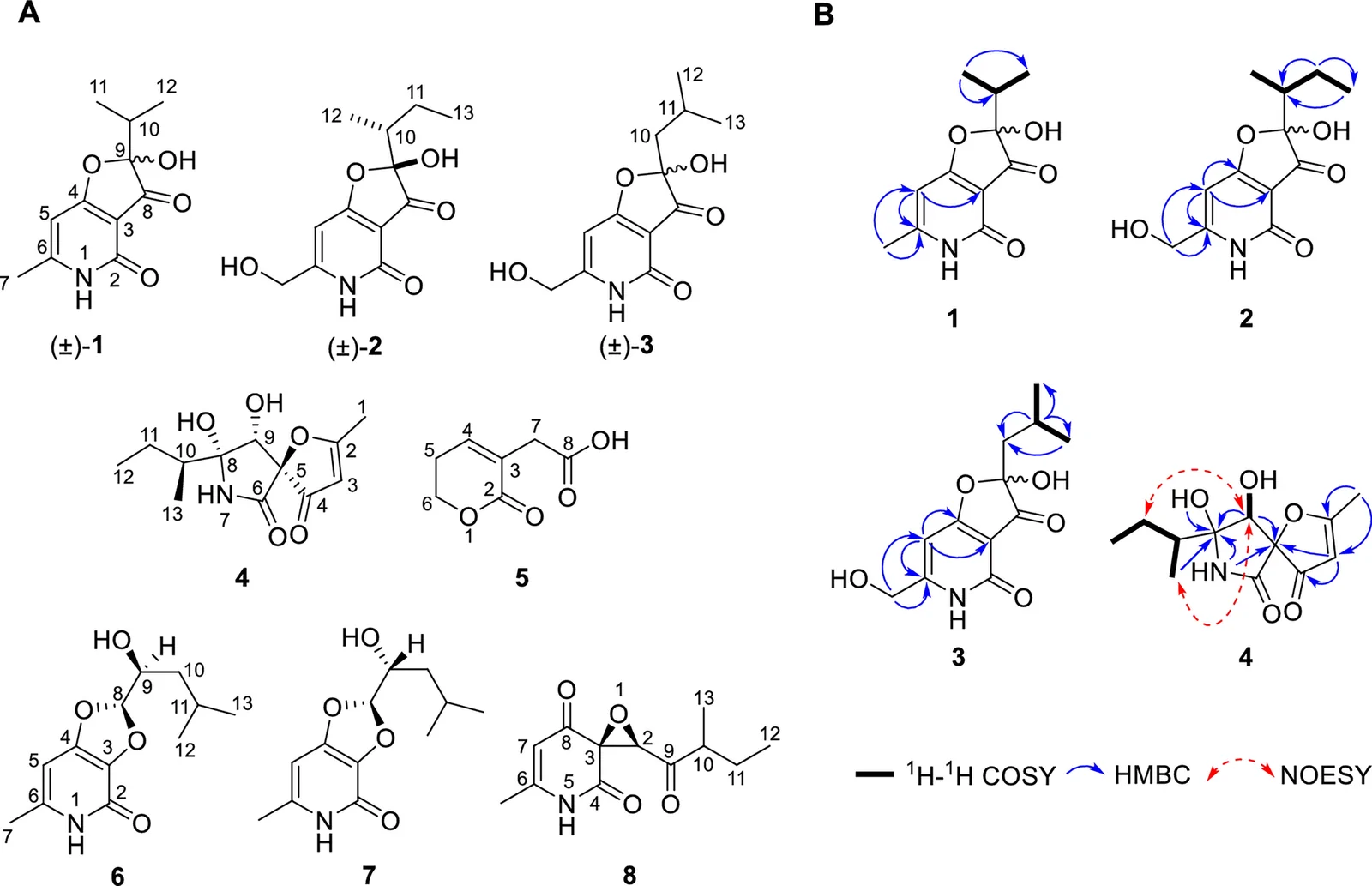
Compounds characterized from *P. negundinis* 12020. **A** Structure of compounds **1**–**8**. **B** Key ^1^H–^1^H COSY, HMBC, and NOESY correlations of compounds** 1**–**4**

**Fig. 3:**
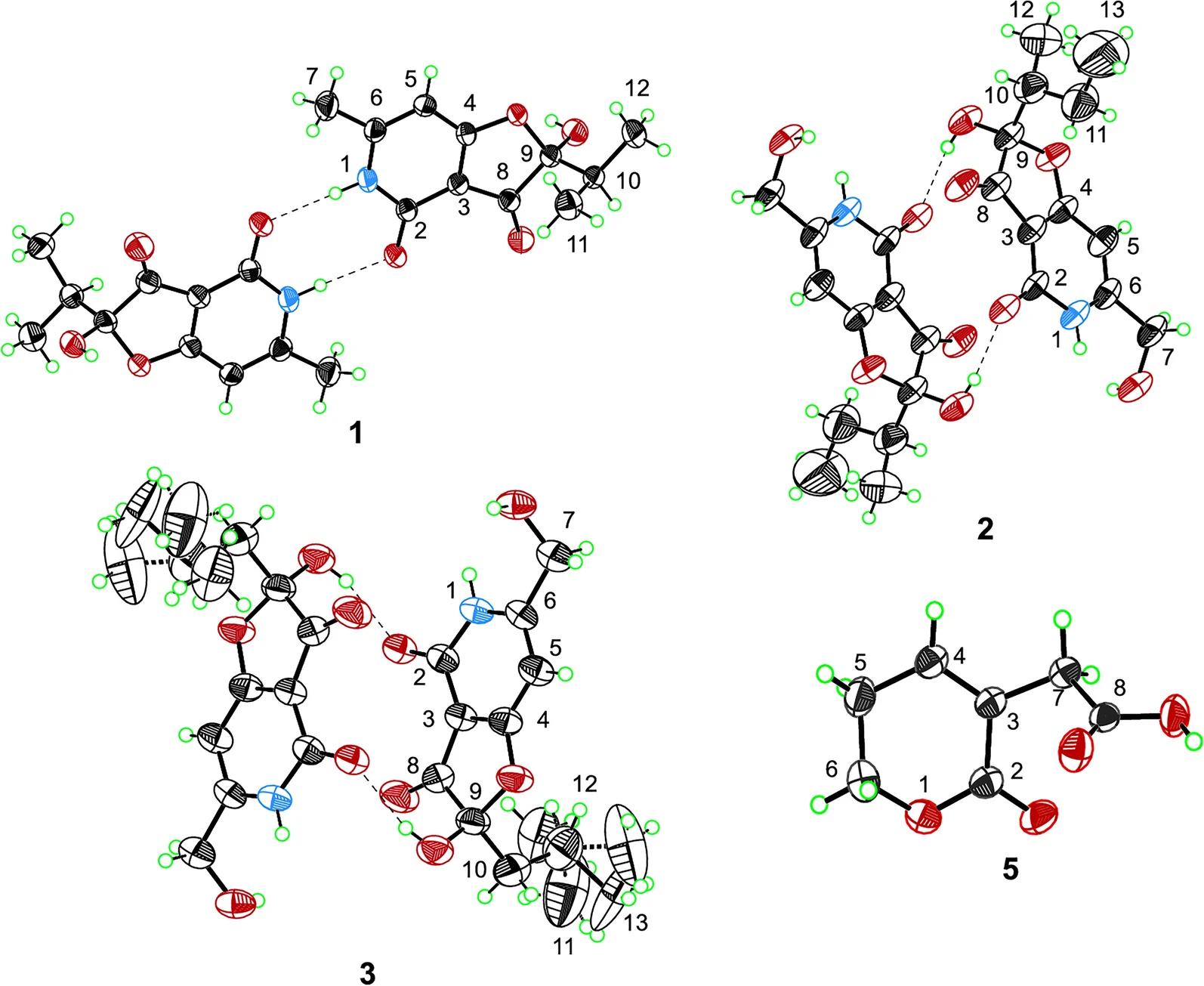
The ORTEP drawing of the X-ray structures of **1**–**3** and **5** with the ellipsoid contours at the 50% probability level

(±)-Phaeopyridone A (**1**) was obtained as a bulk crystal. Its molecular formula was determined as C_11_H_13_NO_4_ through HR-ESI-MS analysis ([M + H]^+^ at *m/z* 224.0918, calcd. for C_11_H_14_NO_4_ 224.0917) (Fig. S4a), indicating six degrees of unsaturation. The UV spectrum showed strong absorption at 320 nm. The ^1^H NMR spectra of **1** (Table 1) displayed three methyl groups [(*δ*_H_ 0.98 (s, H_3_-11), 0.98 (s, H_3_-12), and 2.35 (s, H_3_-10)], one methylene group [*δ*_H_ 2.19 (m, H-11)], and one olefinic proton [*δ*_H_ 6.07 (s, H-5)]. The ^13^C NMR (Table 1) and HSQC (Fig. S4d) spectra of **1** revealed the presence of 10 carbon signals, including three methyls [(*δ*_C_ 16.0 (C-12),* δ*_C_ 16.0 (C-13), and *δ*_C_ 20.1 (C-10)], one methylene (*δ*_C_ 35.3, C-11), four olefinic carbons [(*δ*_C_ 96.6 (C-5),* δ*_C_ 105.1, (C-3),* δ*_C_ 161.1(C-2), and *δ*_C_ 184.4(C-4)], and two carbonyl carbons (*δ*_C_ 161.1, C-2 and *δ*_C_ 196.6, C-8). These functionalities occupied four of six degrees of unsaturation (two double bonds and two carbonyls), suggesting compound **1** has a bicyclic system. The planar structure of compound **1** was further elucidated by 2D NMR spectral data (Fig. 2A). The HMBC correlations (Fig. S4e) from H-5 to C-3, C-4, C-6, and C-7 with H_3_-7 to C-5 and C-6, combined with the higher field carbonyl signal of C-2, revealed the compound **1** contains a 3,4-disubstituted-6-methylpyridin-2(1*H*)-one moiety. The ^1^H–^1^H COSY spectrum (Fig. S4f) revealed the correlations of H-10/H-11 and H-10/H-12 and the HMBC correlations from H_3_-11 to C-10 and C-12 suggested the presence of an isopropyl group -CH(CH_3_)_2_ (Fig. 2B). Although the ^13^C NMR signal and the 2D NMR correlations of C-9 were not detected, the overall structural assignment was ultimately confirmed by single-crystal X-ray diffraction analysis. The bulky crystal of compound **1** was obtained in MeOH/H_2_O (4:1, *v*/*v*) sovlent system at 4 °C, revealing the unprecedented furo[3,2-*c*]pyridone-4 (2*H*)-one skeleton of compound **1** (Fig. 3). The X-ray diffraction data with the triclinic space group *P*1̅, together with the zero specific rotation, demonstrating that **1** existed as a racemic mixture. Nevertheless, repeated attempts to separate pair of enantiomers under various chromatographic conditions and columns were unsuccessful (Fig. S4g).

**Table 1:** NMR Spectroscopic Data (600 MHz for ^1^H NMR, 150 MHz for ^13^C NMR) of compounds **1**–**3**

Pos	**1**		**2**		**3**	
	*δ*_H_ (*J* in Hz)	*δ*_C_, type	*δ*_H_ (*J* in Hz)	*δ*_C_, type	*δ*_H_ (*J* in Hz)	*δ*_C_, type
2		161.1, C		160.8, C		183.9, C
3		105.1, C		105.8, C		104.9, C
4		184.4, C		184.5, C		161.0, C
5	6.07, s	96.6, CH	6.26, s	93.5, CH	6.25, s	93.7, CH
6		159.7, C		162.9, C		163.0, C
7	2.35, s	20.1, CH_3_	4.52, s	61.1, CH_2_	4.53, s	61.1, CH_2_
8		196.6, C		197.0, C		196.7, C
9		n.d		n.d		n.d
10	2.19, m	35.3, CH	1.95, s	42.2, CH	1.87, m	45.2, CH_2_
11	0.98, br s	16.0, CH_3_	1.19, s	23.6, CH_2_	1.88, m	24.4, CH
12	0.98, br s	16.0, CH_3_	0.97, m	12.5, CH_3_	0.96, d (6.5)	24.3, CH_3_
13			0.94, t (7.2)	12.1, CH_3_	0.96, d (6.5)	24.1, CH_3_

Data were recorded in CD_3_OD; “n.d.” means Not detected

(±)-Phaeopyridone B (**2**) was purified as a bulk crystal and assigned a molecular formula of C_12_H_15_NO_5_ with six degrees of unsaturation based on HR-ESI-MS ([M + H]^+^ at *m/z* 254.1033, calcd. for C_12_H_16_NO_5_ 254.1023) (Fig. S5a). Its ^1^H and ^13^C NMR spectrum (Table 1) were similar to compound **1**, except for the absence of a methyl at C-7 and the presence of two methylene in **2** (*δ*_H_ 4.52/*δ*_C_ 61.1, C-7 and *δ*_H_ 1.19/*δ*_C_ 23.6, C-12). The HMBC correlations (Fig. S5e) from H_2_-7 to C-5 and C-6 and from H_3_-13 to C-10 and the ^1^H–^1^H COSY correlations (Fig. S5f) of H_2_-12/H_3_-13 established structural differences between **1** and **2**, which confirmed the planar structure of **2** (Fig. 2). Consequently, the single crystal of **2** was obtained in MeOH-H_2_O (4:1,* v*/*v*) at 4 °C (Fig. 3). The *P*2_1_/*c* space group by single-crystal X-ray diffraction analysis with the zero specific rotation value suggested that **2** was also obatained as a racemic mixture. However, the enantiomerical purity could not be resolved (Fig. S5g).

(±)-Phaeopyridone C (**3**) was isolated as a bulk crystal and assigned a molecular formula of C_12_H_15_NO_5_ with six degrees of unsaturation based on HR-ESI-MS ([M + H]^+^ at *m/z* 254.1032, calcd. for C_12_H_16_NO_5_ 254.1023). Its ^1^H and ^13^C NMR spectrum (Table 1) shared similarity with compound **2**, except for the presence of methylene (*δ*_H_ 4.52/*δ*_C_ 45.2, C-10) in **3**. The HMBC correlations (Fig. S6e) from H-7 to C-5, from H-13 to C-10, and from H-11 to C-10 with the ^1^H–^1^H COSY correlations (Fig. S6f) of H-11/H_3_-12/H_3_-13 established these differences between **1** and **3**, which confirmed the planar structure of **3** (Fig. 2). The single crystal of **3** was obtained in MeOH-H_2_O (4:1,* v*/*v*) at 4 °C (Fig. 3). The *P*2_1_/*c* space group by single-crystal X-ray diffraction analysis with the negligible specific rotation value suggested that **3** was also purified as a racemic mixture but had the same issue for resolution of enantiomeric purity (Fig. S6g).

**Fig. 4:**
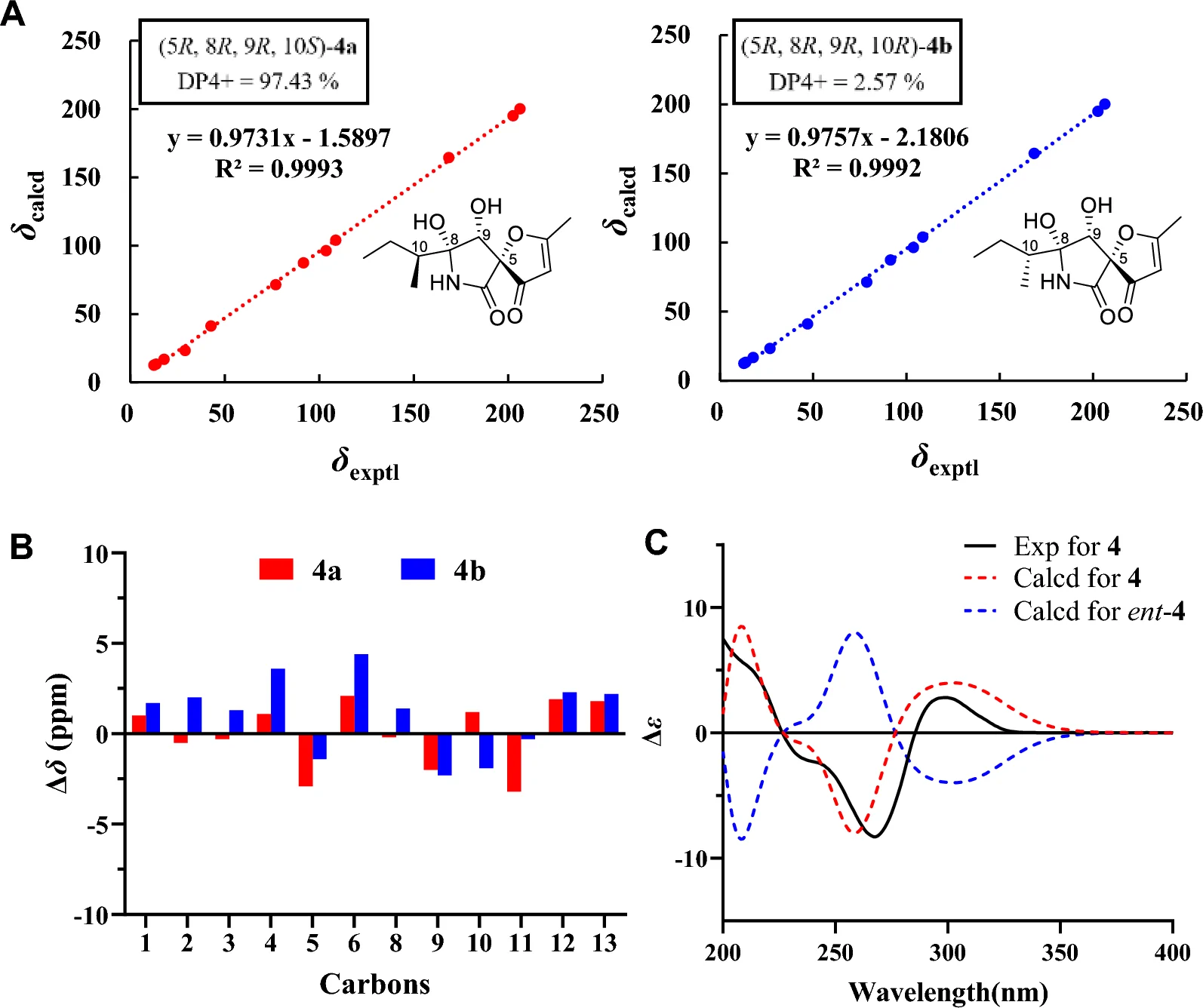
Quantum chemical calculation of two plausible isomers of compound **4**. **A** Linear correlation plots of the calculated and experimental ^13^C NMR values and DP4 + probability. **B** Relative errors between the calculated and recorded ^13^C values. **C** Experimental ECD spectrum of compound **4** in MeOH and the calculated ECD spectra of **4** and *ent*-**4**

Berkeleyamide E (**4**) was obtained as yellow oil, and its molecular formula was determined as C_12_H_17_O_5_N through HR-ESI-MS analysis (*m*/*z* 278.0997 [M + Na]^+^, calcd. for 278.0999, C_12_H_17_O_5_NNa) (Fig. S7a), indicating five degrees of unsaturation. The ^1^H NMR and HSQC spectrum of **4** (Table 2) displayed three methyls [*δ*_H_ 0.85 (t, *J* = 7.3 Hz, H_3_-12), 0.91 (d, *J* = 6.9 Hz, H_3_-13), and 2.36 (s, H_3_-1)], one methylene [*δ*_H_ 1.03 (m, H-11a), 1.69 (m, H-11b)], two *sp*^*3*^ methine [*δ*_H_ 1.62 (d, *J* = 7.3 Hz, H-10) and 4.40 (d, *J* = 7.3 Hz, H-9)], one olefinic proton [*δ*_H_ 5.69 (s, H-3)], and three labile protons [*δ*_H_ 5.37 (8-OH), *δ*_H_ 5.59 (9-OH), *δ*_H_ 9.32 (13-NH)]. The ^13^C NMR (Table 2 and Fig. S7b) spectra of **4** revealed the presence of 12 carbon signals, including three methyls [*δ*_C_ 12.4 (C-12), *δ*_C_ 13.3 (C-13), and *δ*_C_ 16.7 (C-1)], one methylene *δ*_C_ 23.3 (C-11), two *sp*^3^ methine [*δ*_C_ 41.1 (C-10) and *δ*_C_ 71.3 (C-9)], four olefinic carbons [*δ*_C_ 103.9 (C-3), *δ*_C_ 87.3 (C-8), *δ*_C_ 96.3 (C-5), and *δ*_C_ 164.5 (C-6)], and two carbonyl carbons [*δ*_C_ 195.0 (C-2) and *δ*_C_ 200.2 (C-4)]. These signals occupied four of five degrees unsaturation, which suggested compound **4** has a bicyclic system. The planar structure of compound **4** was established further by 2D NMR spectral data (Fig. 2A ). The HMBC correlations (Fig. S7d) from H-1 to C-2/C-3, H-3 to C-4/C-5, H-9 to C-5/C-8, 9-OH to C-5/C-8, 8-OH to C-10/C-8, and 7-NH to C-5/C-8/C-9 revealed the five-membered lactam ring in compound **4**. The HMBC correlations from H-3 to C-4/C-5 further showed spirocyclic structure of compound **4** which was similar to berkeleyamide D [32]. The correlations of H-10/H-11/H-12 and H-10/H-13 displayed in the ^1^H-^1^H COSY (Fig. S7e) spectrum indicated the presence of a *sec*-butyl moiety connected at C-8. The NOESY correlations (Fig. S7f) of H-9/H-11/H-13 suggested the *cis*-vicinal diol at C-9 and C-10, and the comparison with similar structures reported in the literature [33] further confirmed the relative configuration of **4** (Fig. 2B). To determine the absolute configuration at C-5, the ^13^C NMR calculation and DP4 + analysis (Fig. 4A and B) were conducted for the **4a** (10*S*) and **4b** (10*R*) epimers. The result suggested a higher Bayes′s theorem probability for the configuration of **4a** (10*S*) (97.43%) compared to that of **4b** (10*R*) (2.57%) (Table S6). By the chemical calculation using TD-DFT methods, the ECD spectra of **4** was congruent with the experimental data (Fig. 4C), defining the absolute configuration of **4**.

**Table 2:** NMR Spectroscopic Data (600 MHz for ^1^H NMR, 150 MHz for ^13^C NMR) of compounds** 4** and **5**

Pos	**4**^a^		Pos	**5**^b^	
	*δ*_H_ (*J* in Hz)	*δ*_C_, type		*δ*_H_ (*J* in Hz)	*δ*_C_, type
1	2.36, s	16.7, CH_3_	1		
2		195.0, C	2		166.9, C
3	5.69, s	103.9, CH	3		145.4, C
4		200.2, C	4	6.90, s	127.7, CH
5		96.3, C	5	2.51, s	25.4, CH_2_
6		164.5, C	6	4.42, s	68.1, CH_2_
7-NH	9.32, s		7	3.28, s	37.1, CH_2_
8		87.3, C	8		174.7, C
9	4.40, d (5.5)	71.3, CH			
10	1.62, m	41.1, CH			
11a	1.03, m	23.3, CH_2_			
11b	1.69, m				
12	0.85, t (7.3)	12.4, CH_3_			
13	0.91, d (6.9)	13.3, CH_3_			
8-OH	5.37, s				
9-OH	5.95, d (5.6)				

^a^Recorded in DMSO. ^b^Recorded in CD_3_OD

Phaeopyranone (**5**) was obtained as white solid and its molecular formula was determined as C_7_H_8_O_4_ through HR-ESI-MS analysis (*m*/*z* 179.0315 [M + Na]^+^, calcd. for 179.0315, C_7_H_8_O_4_Na), implying four degrees of unsaturation. The ^1^H NMR spectra of **5** (Table 2) displayed three methylenes [*δ*_H_ 2.51 (s), *δ*_H_ 3.82 (s), and *δ*_H_ 4.42 (s)], and one olefinic proton [*δ*_H_ 6.90 (s)]. The ^13^C NMR (Table 2 and Fig. S8b) spectra of **5** suggested seven carbon signals, including three methylene (*δ*_C_ 25.4, 37.1, and 68.1), two olefinic carbons (*δ*_C_ 127.7 and 145.4), and two carbonyl carbons (*δ*_C_ 166.9 and 174.7). The needle crystal of compound **5** was obtained in MeOH/H_2_O (4:1, *v*/*v*) sovlent system at 4 °C. By single-crystal X-ray diffraction analysis, the previously unreported structure of compound **5** was determined (Fig. 3). Additionally, compounds **6**–**8** were determined as (8*S*,9*S*)-dihydroisoflavipucine (**6**) [30], (8*S*,9*R*)-dihydroisoflavipucine (**7**) [31], and sapinopyridione (**8**) [31] by comparing spectroscopic data in previous literature.

Compounds **1**–**3** and **6**–**7** were evaluated for the antimicrobial activities against *C*. *albicans* ATCC10231, *E. coli* ATCC11775, and *S. aureus* ATCC6538. Additionally*,* Compounds **1**–**4** and **6**–**7** were evaluated for the antimicrobial activities against *E*. *tarda* ATCC15947, *B*. *cereus* ATCC14579, *A*. *baumannii* ATCC19606, and *E*. *aerogenes* ATCC13048. However, none of them displayed antimicrobial activity against these pathogens. In contrast, compound **5** showed anti-tuberculosis activity with an MIC of 6.25 μg/mL when all compounds were evaluated for anti-tuberculosis activity against *M. tuberculosis* (Table S7). 2-Pyridone derivatives are known to exhibit a broad range of biological activity, including cytotoxic, antibacterial, antiviral activity, and so on, which are considered privileged scaffolds in medicinal chemistry [1]. Therefore, the newly identified furo[3,2-c]pyridone scaffold may possess additional unexplored activities. Further evaluation against diverse biological targets is warranted to fully assess its pharmacological potential.

### Phylogenetic analysis of Orf12 and proposed biosynthesis pathway of flavipucine alkaloids

**Fig. 5:**
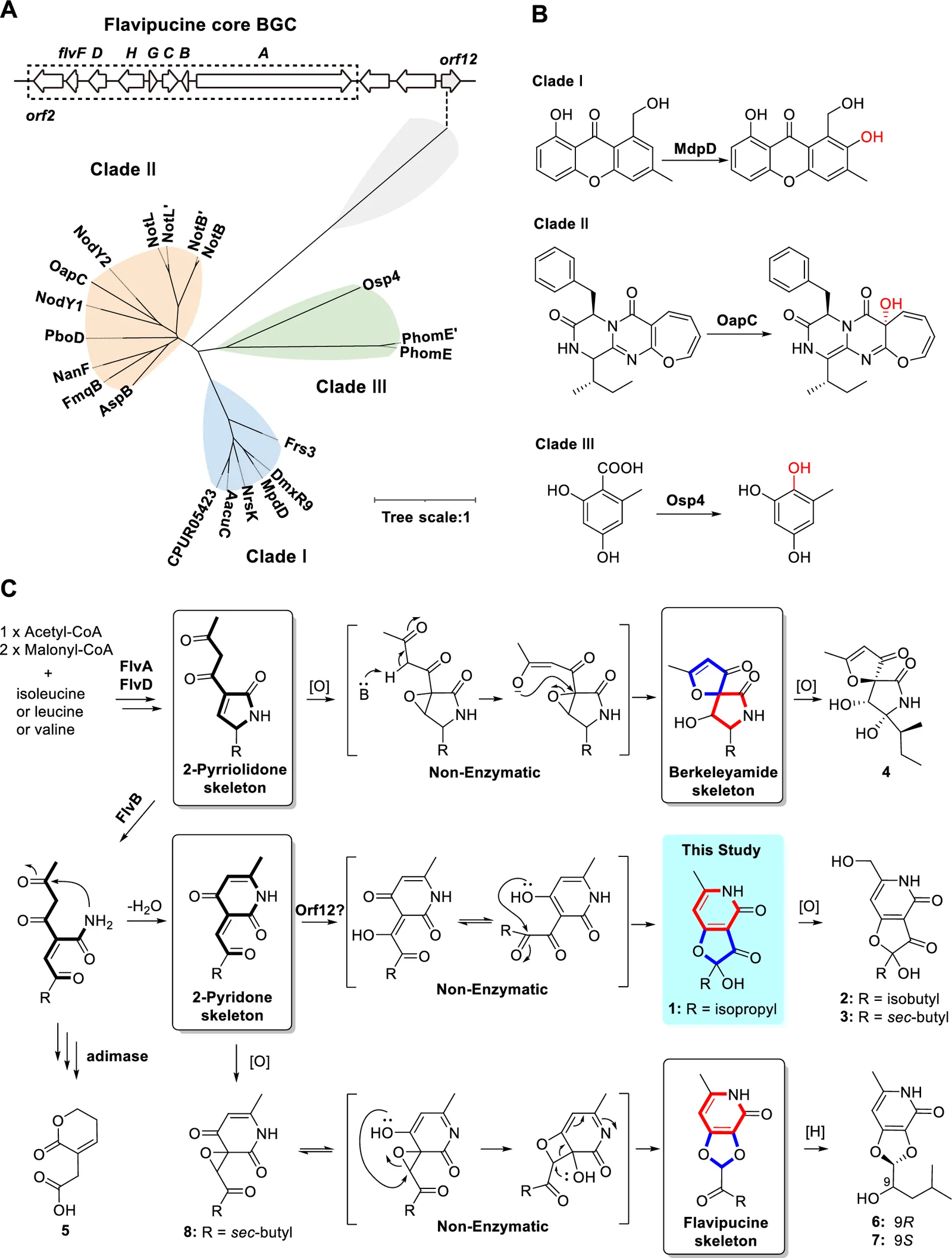
Enzyme phylogeny analysis and plausible biosynthesis pathway. **A** Phylogenetic analysis Orf12 with homologues from BLASTp search. **B** Representative catalytic reactions of enzymes from different clades. **C** Proposed biosynthesis pathway of flavipucine and derivatives

A phylogenetic analysis was undertaken to speculate the potential functions of Orf12. This analysis provides valuable insights into the evolutionary relationship between Orf12 and FAD-dependent monooxygenases through BLASTp search (Fig. 5A and Table S8). The phylogenetic tree comprises four distinct clades, with Orf12 forming its own unique clade. Among them, enzymes within Clade I and III are responsible for catalyzing the hydroxylation of aromatic rings in natural products including xanthone and naphthoquinones [29, 34–39]. Furthermore, enzymes in Clade II are responsible for the hydroxylation and epoxidation in nitrogen-containing heterocyclic natural product BGCs [40–45] (Fig. 5B and Fig. S12). Based on the result of phylogenetic analysis, it is hypothesized that Orf12 shares a closer phylogenetic relationship with Clade II and may catalyze the hydroxylation of nitrogen-containing heterocyclic natural products, such as 2-pyridones.

The biosynthesis of 2-pyridone have been elucidated previously: ʟ-leucine, ʟ-isoleucine, or ʟ-valine, in conjunction with two molecules of malonyl-CoA, serve as substrates for FlvAD, which catalyze the formation of a 2-pyrrolidone intermediate. This intermediate subsequently undergoes C–N bond cleavage mediated by FlvB, followed by spontaneous cyclization to yield the 2-pyridone scaffold [17]. Both the 2-pyrrolidone and 2-pyridone scaffolds, following a series of oxidation processes, undergo non-enzymatic intramolecular cyclization, leading to the formation of distinct cyclic scaffolds. Specifically, the enzymatically catalyzed epoxidation product of the 2-pyrrolidone scaffold undergoes further non-enzymatic intramolecular cyclization to the formation of a spirocyclic structure, which serves as the precursor to compound **4**. Meanwhile, the 2-pyridone scaffold is subject to both enzymatic epoxidation and hydroxylation. The hydroxylation reaction in this step is likely catalyzed by Orf12. Under acidic conditions, the epoxy product of the 2-pyridone scaffold undergoes an intermolecular nucleophilic attack by the carbonyl oxygen, resulting in epoxide ring opening. The resulting oxetane intermediate then experiences a subsequent nucleophilic attack triggered by a tert-hydroxy function, which is followed by C–C bond cleavage to yield a five-membered acetal fused to the 2-pyridone rings which is the precursor to compounds **6** and **7** [46]. In contrast, the hydroxylated product of the 2-pyridone scaffold, after keto-enol tautomerism, forms a hemiacetal structure through nucleophilic attack by the hydroxyl group. This transformation results in the formation of the furo[3,2-*c*]pyridone-4 (2*H*)-one scaffold, which is the precursor to compounds **1**–**3** (Fig. 5C).

Natural products biosynthesis is primarily driven by enzyme-catalyzed systems, in which sets of tailoring enzymes introduce complexity of natural products by covalently modifying functional groups in pathway intermediates [47]. In this research, comparative and detailed analysis of the gene clusters revealed a non-conserved FAD-dependent monooxygenase within the flavipucine BGC of *P. negundinis* 12020 and a cytochrome P450 within the flavipucine BGC of *H. roseus* 17014. Although not universally conserved, these enzymes may be a key factor in the formation of novel skeletal structures observed in this strain. Compared with the previously reported biosynthetic pathways, this proposed hydroxylation reaction enables the subsequent non-enzymatic nucleophilic attack, leading to formation of unique furo[3,2-*c*]pyridones scaffolds and expansion of structural diversity. Although the bioinformatic evidence supports the proposed function of FAD-dependent monooxygenase Orf12, definitive validation will require future in vivo gene knockout experiments and/or in vitro enzymatic assays.

Despite the dominant role of enzymatic catalysis in natural product biosynthesis, there are instances in which the inherent chemical reactivity of metabolic intermediates and substrates leads to reactions occurring independently of enzymatic catalysis. Non-enzymatic processes also play a significant role in shaping the structural diversity of natural products [48–50]. Though less common and challenging to characterize, these non-enzymatic reactions, particularly nucleophilic attacks, play an important role in post-modification of distinct and complex scaffolds and expansion of their chemical space. This highlights the need to consider both enzymatic and non-enzymatic processes to fully grasp the biosynthetic potential of natural products.

## Conclusion

In summary, genome mining and bioinformatic analysis led to the identification of the flavipucine BGC in *P. negundinis* 12020. FBMN analysis has revealed the presence of flavipucine-related alkaloids, and HR-MS-guided isolation has contributed to the characterization of five novel compounds (**1**–**5**). Notably, compounds **1**–**3** possess an unprecedented furo[3,2-*c*]pyridone core structure. The structures of these compounds were unambiguously confirmed through integrated MS, NMR, and X-ray diffraction data analysis. Though the biosynthesis of flavipucine alkaloids has been previously studied, an FAD-dependent monooxygenase was in the flavipucine BGC of *P. negundinis* 12020 may lay the foundation for the formation of hemiacetal structures which requires further biochemical validation. The biological activities of these compounds remain to be further investigated. This research not only expands the known flavipucine family of molecules but also highlights the underexplored potentiality of endophytic fungi as sources of novel compounds.

## Supplementary Information

Supplementary material 1.

## Data Availability

Data will be made available on request.
