# Correction: Genome mining guided identification of new furo[3,2‑*c*]pyridones phaeopyridones A–C from the endophytic fungus *Phaeobotryon negundinis* 12020

**DOI:** 10.1007/s13659-026-00666-4

**Published:** 2026-10-30

**Authors:** Ke Ye, Jie Shi, Huimin Wu, Shujie Wang, Zexu Lin, Jiale Sun, Yuwei Chen, Bohao Wang, Rongxuan Wang, Chenglin Jiang, Yi Jiang, Tom Hsiang, Bin Zhu, Lixin Zhang, Xueting Liu, Guoliang Zhu

**Affiliations:** 1 State Key Laboratory of Bioreactor Engineering, East China University of Science and Technology, Shanghai, 200237 China; 2 Nanjing Technology University, Nanjing, 211816 China; 3 The Key Lab for Research and Development of Actinomycete Resources, Yunnan Institute of Microbiology, Yunnan University, Chenggong District, Kunming, 650500 China; 4 School of Environmental Sciences, University of Guelph, Guelph, ON N1G 2W1 Canada

**Correction: Natural Products and Bioprospecting (2026) 16:85**

**https://doi.org/10.1007/s13659-026-00644-w**

Following publication of the original article [1], the author found that the places of Figs. 2 and 3 should be switched.

The updated version of Figs. 2 and 3 is:

**Fig. 2:**
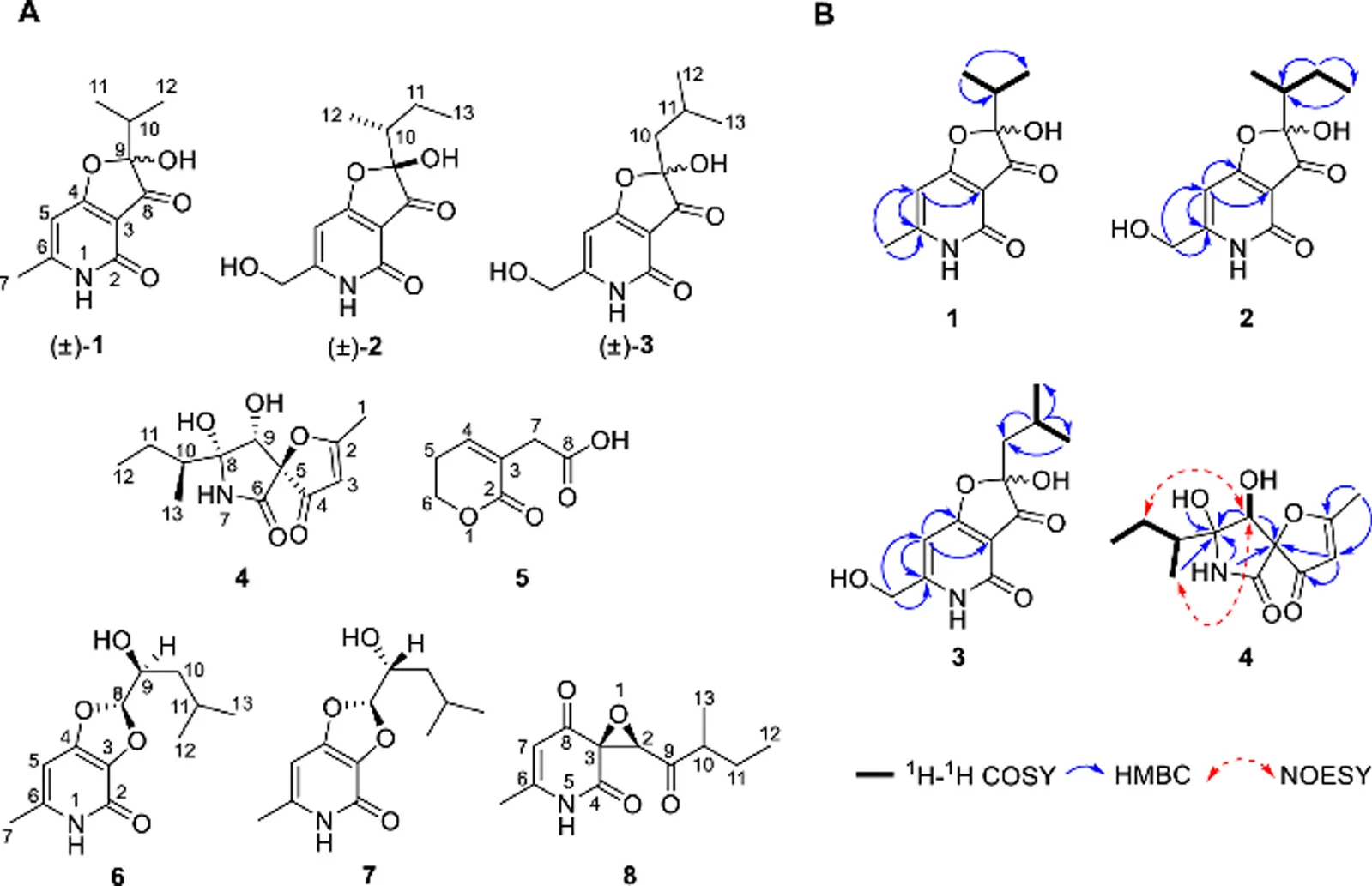
Compounds characterized from *P. negundinis* 12020.** A** Structure of compounds** 1**–**8**.** B** Key 1H–1H COSY, HMBC, and NOESY correlations of compounds** 1**–**4**

**Fig. 3:**
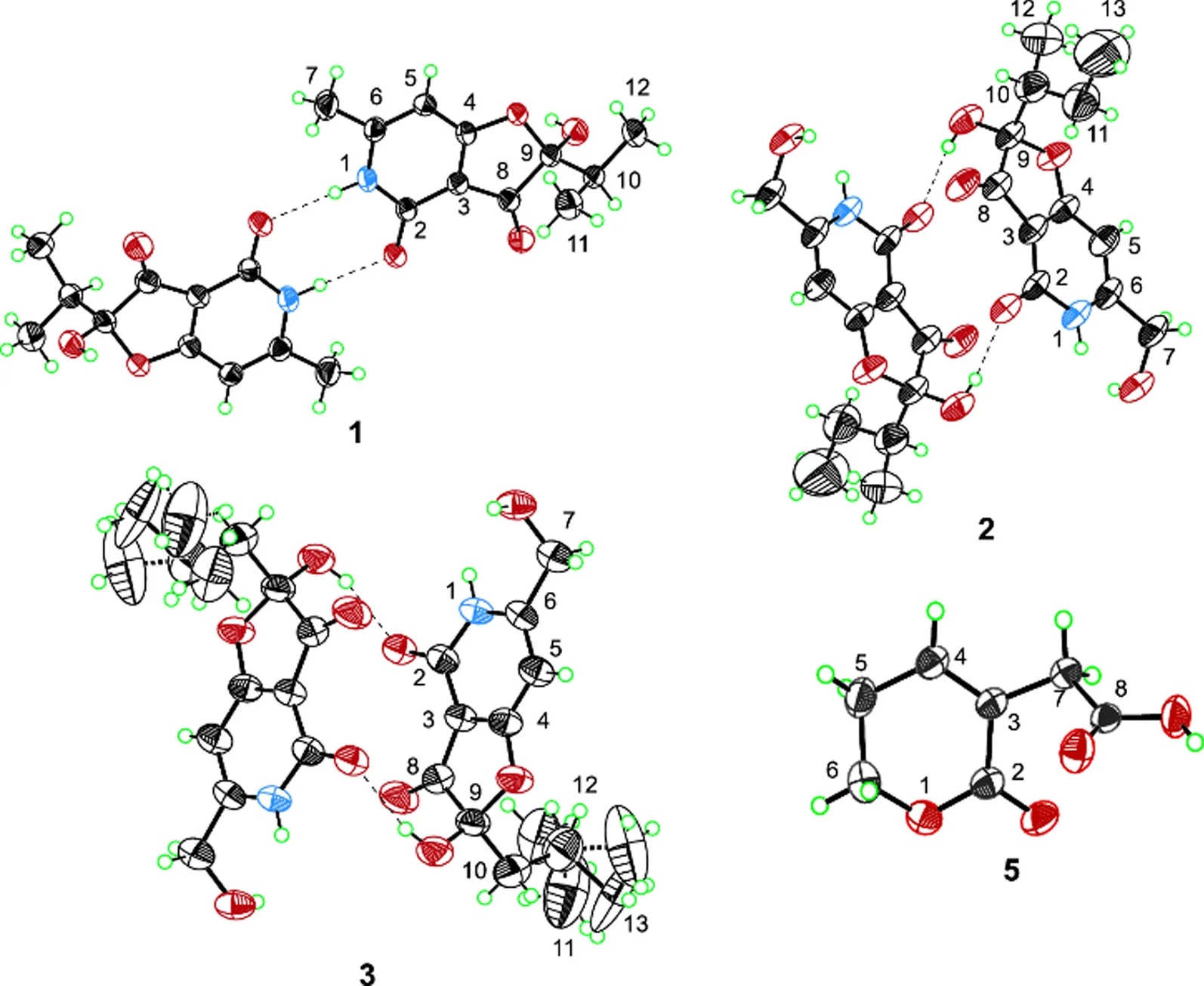
The ORTEP drawing of the X-ray structures of** 1**–**3** and** 5** with the ellipsoid contours at the 50% probability level

The original article [1] has been updated.
