# Supplementary material for: Genome mining guided identification of new furo[3,2-c]pyridones phaeopyridones A–C from the endophytic fungus Phaeobotryon negundinis 12020
Source: Nat Prod Bioprospect. 2026 Aug 28;16(1):85. doi: 10.1007/s13659-026-00644-w (PMC13525150; doi:10.1007/s13659-026-00644-w)
Supplement: Supplementary file 1 [file 13659_2026_644_MOESM1_ESM.docx]

**Supporting Information**

**Genome Mining Guided Identification of New Furo[3,2-*c*]pyridones Phaeopyridones A−C from the Endophytic Fungus *Phaeobotryon negundinis* 12020**

Ke Ye^a^, Jie Shi^a^, Huimin Wu^a^, Shujie Wang^a^, Zexu Lin^a^, Jiale Sun^a^, Yuwei Chen^b^, Bohao Wang^a^, Rongxuan Wang^a^, Chenglin Jiang^c^, Yi Jiang^c^, Tom Hsiang^d^, Bin Zhu^a^, Lixin Zhang^a^, Xueting Liu^a^, Guoliang Zhu^a,^*

^a^State Key Laboratory of Bioreactor Engineering, East China University of Science and Technology, Shanghai 200237, China

^b^Nanjing Technology University, Nanjing 211816, China

^c^The Key Lab for Research & Development of Actinomycete Resources, Yunnan Institute of Microbiology, Yunnan University, Chenggong District, Kunming 650500, China.

^d^School of Environmental Sciences, University of Guelph, Guelph, Ontario N1G 2W1, Canada

*Corresponding author: Guoliang Zhu

E-mail: zhuguoliang@ecust.edu.cn

Phone (Fax): CN +86-021-64253020.

Table of Contents

[Supplementary Texts	5](#_Toc219909133)

[1. The ITS region of the fungus Phaeobotryon negundinis 12020	5](#_Toc219909134)

[2. General experimental procedures	5](#_Toc219909135)

[3. GNPS directed dereplication and prediction of new flavipucine alkaloids	6](#_Toc219909136)

[4. Quantum chemical calculations	7](#_Toc219909137)

[5. Spectral data of compounds 1−8	7](#_Toc219909138)

[Supplementary Figures	10](#_Toc219909139)

[Fig. S1 Identification of strain *P. negundinis* 12020.	10](#_Toc219909140)

[Fig. S2 Overview of the molecular networking generated from LC-MS/MS dataset of strain *P. negundinis* 12020.	11](#_Toc219909141)

[Fig. S3 Secondary MS fragmentation analysis of compounds 1−4 and 6−8.	12](#_Toc219909142)

[Fig. S4a HR-ESI (Pos)-MS spectrum of (±)-phaeopyridone A (1).	13](#_Toc219909143)

[Fig. S4b ^1^H NMR (600 MHz, CD_3_OD) spectrum of (±)-phaeopyridone A (1).	13](#_Toc219909144)

[Fig. S4c ^13^C NMR spectrum (150 MHz, CD_3_OD) of (±)-phaeopyridone A (1).	14](#_Toc219909145)

[Fig. S4d HSQC spectrum of (±)-phaeopyridone A (1).	14](#_Toc219909146)

[Fig. S4e HMBC spectrum of (±)-phaeopyridone A (1).	15](#_Toc219909147)

[Fig. S4f ^1^H−^1^H COSY spectrum of (±)-phaeopyridone A (1).	15](#_Toc219909148)

[Fig. S4g The chiral separation result of (±)-phaeopyridone A (1).	16](#_Toc219909149)

[Fig. S5a HR-ESI (Pos)-MS spectrum of (±)-phaeopyridone B (2).	17](#_Toc219909150)

[Fig. S5b ^1^H NMR spectrum (600 MHz, CD_3_OD) of (±)-phaeopyridone B (2).	17](#_Toc219909151)

[Fig. S5c ^13^C NMR spectrum (150 MHz, CD_3_OD) of (±)-phaeopyridone B (2).	18](#_Toc219909152)

[Fig. S5d HSQC spectrum of (±)-phaeopyridone B (2).	18](#_Toc219909153)

[Fig. S5e HMBC spectrum of (±)-phaeopyridone B (2).	19](#_Toc219909154)

[Fig. S5f ^1^H−^1^H COSY spectrum of (±)-phaeopyridone B (2).	19](#_Toc219909155)

[Fig. S5g The chiral separation result of (±)-phaeopyridone B (2).	20](#_Toc219909156)

[Fig. S6a HR-ESI (Pos)-MS spectrum of (±)-phaeopyridone C (3).	21](#_Toc219909157)

[Fig. S6b ^1^H NMR spectrum (600 MHz, CD_3_OD) of (±)-phaeopyridone C (3).	21](#_Toc219909158)

[Fig. S6c ^13^C NMR spectrum (150 MHz, CD_3_OD) of (±)-phaeopyridone C (3).	22](#_Toc219909159)

[Fig. S6d HSQC spectrum of (±)-phaeopyridone C (3).	22](#_Toc219909160)

[Fig. S6e HMBC spectrum of (±)-phaeopyridone C (3).	23](#_Toc219909161)

[Fig. S6f ^1^H−^1^H COSY spectrum of (±)-phaeopyridone C (3).	23](#_Toc219909162)

[Fig. S6g The chiral separation result of (±)-phaeopyridone C (3).	24](#_Toc219909163)

[Fig. S7a HR-ESI (Pos)-MS spectrum of berkeleyamide E (4).	25](#_Toc219909164)

[Fig. S7b ^1^H NMR (600 MHz, DMSO) spectrum of berkeleyamide E (4).	25](#_Toc219909165)

[Fig. S7c ^13^C NMR (150 MHz, DMSO) spectrum of berkeleyamide E (4).	26](#_Toc219909166)

[Fig. S7d HSQC spectrum of berkeleyamide E (4).	26](#_Toc219909167)

[Fig. S7e HMBC spectrum of berkeleyamide E (4).	27](#_Toc219909168)

[Fig. S7f ^1^H−^1^H COSY spectrum of berkeleyamide E (4).	27](#_Toc219909169)

[Fig. S7g NOESY spectrum of berkeleyamide E (4).	28](#_Toc219909170)

[Fig. S8a HR-ESI (Pos)-MS spectrum of phaeopyranone (5).	29](#_Toc219909171)

[Fig. S8b ^1^H NMR (600 MHz, CD_3_OD) spectrum of phaeopyranone (5).	29](#_Toc219909172)

[Fig. S8c ^13^C NMR (150 MHz, CD_3_OD) spectrum of phaeopyranone (5).	30](#_Toc219909173)

[Fig. S9a HR-ESI (Pos)-MS spectrum of (8*S*,9*S*)-dihydroisoflavipucine (6).	31](#_Toc219909174)

[Fig. S9b ^1^H NMR (600 MHz, CD_3_OD) spectrum of (8*S*,9*S*)-dihydroisoflavipucine (6).	31](#_Toc219909175)

[Fig. S9c ^13^C NMR (150 MHz, CD_3_OD) spectrum of (8*S*,9*S*)-dihydroisoflavipucine (6).	32](#_Toc219909176)

[Fig. S10a HR-ESI (Pos)-MS spectrum of (8*S*,9*R*)-dihydroisoflavipucine (7).	33](#_Toc219909177)

[Fig. S10b ^1^H NMR (600 MHz, CD_3_OD) spectrum of (8*S*,9*R*)-dihydroisoflavipucine (7).	33](#_Toc219909178)

[Fig. S10c ^13^C NMR (150 MHz, CD_3_OD) spectrum of (8*S*,9*R*)-dihydroisoflavipucine (7).	34](#_Toc219909179)

[Fig. S11a HR-ESI (Pos)-MS spectrum of sapinopyridione (8).	35](#_Toc219909180)

[Fig. S11b ^1^H NMR (600 MHz, CD_3_OD) spectrum of sapinopyridione (8).	35](#_Toc219909181)

[Fig. S11c ^13^C NMR (150 MHz, CD_3_OD) spectrum of sapinopyridione (8).	36](#_Toc219909182)

[Fig. S12 The reactions catalyzed by homologues of Orf12.	37](#_Toc219909183)

[Supplementary Tables	38](#_Toc219909184)

[Table S1. Enzyme function annotation of BGC of *Hypomyces roseus* 17014.	38](#_Toc219909185)

[Table S2. Enzyme function annotation of BGC of *P. negundinis* 12020.	39](#_Toc219909186)

[Table S3. Annotation for 22 nodes in the pyridone clusters I&II from the PN12020 Featured Based Molecular Networking.	40](#_Toc219909187)

[Table S4. Crystal data and structure refinements for (±)-phaeopyridone A−C (1−3), and phaeopyranone (5).	41](#_Toc219909188)

[Table S5. NMR Spectroscopic Data (CD_3_OD, 600 MHz for ^1^H, 150 MHz for ^13^C) of (8*S*,9*S*)-dihydroisoflavipucine (6), (8*S*,9*R*)-dihydroisoflavipucine (7), and sapinopyridione (8).	42](#_Toc219909189)

[Table S6. DP4 probability of ^13^C NMR chemical shifts of 4a (5*R**,8*R**,9*R**,10*S**) and 4b (5*R**,8*R**,9*R**,10*R**).	43](#_Toc219909190)

[Table S7. Antimicrobial activity of compounds 1−7.	44](#_Toc219909191)

[Table S8. The sequences selected for the phylogenetic analysis.	45](#_Toc219909192)

# Supplementary Texts

## 1. The ITS region of the fungus *Phaeobotryon negundinis* 12020

CTTGTAAGGTGACTGCGGAGACATTACCGAGTTCTCGGGCTTCGGCTCGAATCTCCCACCCTTTGTGAACATACCTCTGTTGCTTTGGCGGCTCGAGAGCGCCCCGTGCGATCGCGACCGCCAGAGGACCTTAAAACTCCAGTCAGTAAACGTCTACGTCTGATAAACAAGTTAATAAACTAAAACTTTCAACAACGGATCTCTTGGTTCTGGCATCGATGAAGAACGCAGCGAAATGCGATAAGTAATGTGAATTGCAGAATTCAGTGAATCATCGAATCTTTGAACGCACATTGCGCCCCTTGGTATTCCGAGGGGCATGCCTGTTCGAGCGTCATTACAACCCTCAAGCTCTGCTTGGTATTGGGCGTCGTCCTCCTGCGGACGCGCCTCAAAGACCTCGGCGATGGCGTCTAGCCCTCAAGCGTAGTAGAATACACCTCGCTTTGGAGCGGTTGGCGTCGTCCGCCGGACGAACCTTCTGAACTTTTCTCAAGGTTGACCTCGGATCAGGTAGGGATACCCGCTGAACTTAAGCATATCAAAAGGCCGGAGGAAA

2. **General experimental procedures**

Nuclear magnetic resonance (NMR) spectra were measured on an Avance Neo 600 MHz spectrometer (600 MHz for ^1^H NMR and 150 MHz for ^13^C NMR) (Bruker, Billerica, MA, USA) and VNMRS 600 MHz spectrometer (600 MHz for ^1^H NMR and 150 MHz for ^13^C NMR) (Varian). ^1^H NMR chemical shifts were reported in *δ* value based on residual signal from CD_3_OD (3.31 and 4.87 ppm) and dimethyl sulfoxide-*d_6_* (2.50 and 3.30 ppm) as reference. ^13^C NMR chemical shifts were reported in *δ* value based on residual signal from CD_3_OD (49.0 ppm) and dimethyl sulfoxide-*d_6_* (39.5 ppm) as a reference. High-resolution electrospray ionization mass spectrometry (HR-ESI-MS) measurements were performed using Thermo Q Exactive Orbitrap mass spectrometer coupled to a Shimadzu LC-30AD UPLC which was equipped with a Waters ACQUITY BEH C18 column (2.1 × 100 mm, 1.7 μm particles). Specific rotation measurements were conducted on a Perkin Elmer Model 343 polarimeter (Waltham, MA, USA) with samples in methanol solution. CD spectra of isolated compounds were record using chirascan circular dichroism spectrometer with methanol as solvent. X-ray crystallographic analysis was conducted on a Bruker APEX-III CCD diffractometer (Bruker, Billerica, MA, USA). The LC-MS analysis was run using an Agilent 1260 Series separation module with an Agilent InfinityLab LC/MSD iQ MS detector. Sephadex LH-20 was utilized for column chromatography (CC). High-performance liquid chromatography (HPLC) was conducted using an Agilent 1260 HPLC system equipped with a diode array detector. Preparative HPLC was performed using an ACE C18-PFP column (21.2 × 250 mm, 5 μm) and a Basic C18 column (21.2 × 250 mm, 5 μm). Semipreparative HPLC was performed using a Morphing C18T column (10 × 250 mm, 5 μm) and a ZORBAX SB-C18 column (10 × 250 mm, 5 μm). Chiral separation was attempted using several chiral stationary phases, including Daicel Chiralpak AD-H, AS-H, OD-H, OJ-H, IA, IB, and IC columns, under mobile phase conditions composed of n-hexane/ethanol (90:10, 85:15, 80:20, 70:30, and 65:35, *v*/*v*). All solvents used were of HPLC/LCMS grade.

3. **GNPS directed dereplication and prediction of new flavipucine alkaloids**

Samples were dissolved in MeOH to make 1 mg/mL solution and eluted with a gradient of H_2_O (1‰ HCOOH) and CH_3_CN with a gradient method as follows: 5% CH_3_CN gradient elution to 30% in 9 min, 99% CH_3_CN kept for 3 min, 99% CH_3_CN changed to 5% in 0.1 min, and 5% CH_3_CN kept for 3 min with the flow rate of 0.35 mL/min. LC-MS/MS data was acquired on Thermo Q Exactive Orbitrap mass spectrometer coupling with a Shimadzu LC-30AD UPLC system. The raw data was converted to the mzXML file format using the Bruker Data Analysis software. The mzML file was processed using the MZmine (version 2.53). The mass detections were realized keeping the noise level at 1.0E^7^ for MS1 and 1.0E^4^ for MS2, respectively. The ADAP chromatogram building used a minimum time span of 0.1 min, *m*/*z* tolerance of 0.02 (or 5 ppm), and minimum height of 3.0E^7^. Deisotope was carried out utilizing isotopic peaks grouper algorithm, with a RT tolerance of 0.1 min and *m*/*z* tolerance of 0.01 (or 5 ppm). Duplicate peaks were filtered with filter mode of old average, *m*/*z* tolerance of 0.01 (or 5 ppm), and retention time (RT) range = 1 min. A feature-based molecular networking (FBMN) was created using the web-based workflow (version release_28.2) at GNPS [1]. Cytoscape (version 3.7.2) was used to visualize the corresponding output FBMN data.

4. **Quantum chemical calculations**

Density functional theory (DFT) was used for the computations applied in Gaussian 09 [2]. A conformational search of all possible isomers was conducted using the Merck Molecular Force Field (MMFF) 94 in Sybyl X 2.0 software, with an energy cutoff value of 3 kcal/mol and a random search algorithm. Ground-state geometry was optimized at the level of B3LYP/6-31G(d). ^13^C NMR calculation was conducted by using B3LYP/6-311+G (2d, p) level. [Solvent effects](https://www.sciencedirect.com/topics/biochemistry-genetics-and-molecular-biology/solvent-effect) of methanol solution were evaluated at the same DFT level by using the IEF/PCM method. TD-DFT was performed by using B3LYP/6-311G (d) level in methanol to calculate the [electronic excitation](https://www.sciencedirect.com/topics/chemistry/electronic-excitation) energies and rotational strengths.

5. **Spectral data of compounds 1−8**

(±)-Phaeopyridone A (**1**): Bulky crystals; UV (MeOH) *λ*_max_ (log *ε*) 235 (4.28), 274 (3.77), 321 (4.42) nm; HR-ESI-MS *m/z* 224.0918 [M + H]^+^ (calcd. for C_11_H_14_NO_4_ 224.0917) (Fig. S4a); ^1^H and ^13^C NMR data, see Table 1 and Figs. S4b−S4f.

(±)-Phaeopyridone B (**2**): Bulky crystals; UV (MeOH) *λ*_max_ (log *ε*) 233 (4.27), 276 (3.61), 320 (4.33) nm; HR-ESI-MS *m/z* 254.1033 [M + H]^+^(calcd. for C_12_H_16_NO_5_ 254.1023) (Fig. S5a). ^1^H and ^13^C NMR data, see Table 1 and Figs. S5b−S5f.

(±)-Phaeopyridone C (**3**): Bulky crystals; UV (MeOH) *λ*_max_ (log *ε*) 233 (4.45), 275 (3.85), 320 (4.50) nm; HR-ESI-MS *m/z* 254.1032, [M + H]^+^ (calcd. for C_12_H_16_NO_5_ 254.1023) (Fig. S6a); ^1^H and ^13^C NMR data, see Table 1 and Figs. S6b−S6f.

Berkeleyamide E (**4**): Yellow oil; HR-ESI-MS *m/z* 278.0997 [M + Na]^+^ (calcd. for C_12_H_17_NO_5_ 278.0999) (Fig. S7a); UV (MeOH) *λ*_max_ (log *ε*) 271 (3.89) nm; ^1^H and ^13^C NMR data, see Table 2 and Figs. S7b−S7g.

Phaeopyranone C (**5**): Needle crystals; UV (MeOH) *λ*_max_ (log *ε*) 216 (4.20) nm; HR-ESI-MS *m/z* 179.0315 [M + Na]^+^ (calcd. for C_7_H_8_O_4_Na 179.0315) (Fig. S8a); ^1^H and ^13^C NMR data, see Table 2 and Figs. S8b−S8c.

(8*S*,9*S*)-Dihydroisoflavipucine (**6**): [*α*]25 D = + 231 (*c* 0.1, MeOH); UV (MeOH) *λ*_max_ (log *ε*) 219 (4.72), 252 (3.93), 306 (4.44) nm; HR-ESI-MS *m/z* 240.1234, [M + H]^+^ (calcd. for C_12_H_18_NO_4_ 240.1230) (Fig. 9a); ^1^H and ^13^C NMR data, see Table S4 and Figs. S9b−S9c.

(8*S*,9*R*)-Dihydroisoflavipucine (**7**): [*α*]25 D = - 6 (*c* 0.1, MeOH); UV (MeOH) *λ*_max_ (log *ε*) 217 (4.75), 252 (3.81), 306 (4.32) nm; HR-ESI-MS *m/z* 240.1233, [M + H]^+^ (calcd. for C_12_H_18_NO_4_ 240.1230) (Fig. S10a); ^1^H and ^13^C NMR data, see Table S4 and Figs. S10b−S10c.

Sapinopyradione (**8**): bulky crystals; [*α*]25 D = - 15 (*c* 0.1, MeOH); UV (MeOH) *λ*_max_ (log *ε*) 226 (4.30), 275 (3.23), 319 (4.14) nm; HR-ESI-MS *m/z* 238.1066, [M + H]^+^ (calcd. for C_12_H_16_NO_4_ 238.1074) (Fig. S11a); ^1^H and ^13^C NMR data, see Table S4 and Figs. S11b−S11c.

**Reference**

[1]	Nothias LF, Petras D, Schmid R, Dührkop K, Rainer J, Sarvepalli A, et al. Feature-based molecular networking in the GNPS analysis environment. Nat Methods 2020;17(9):905-8. <http://doi.org/10.1038/s41592-020-0933-6>.

[2]	Frisch MJ, Trucks GW, Schlegel HB, Scuseria GE, Robb MA, Cheeseman JR, et al. Gaussian 09 Rev. A.02 . Wallingford, CT. 2016.

# Supplementary Figures

## Fig. S1 Identification of strain *P. negundinis* 12020.

(**A**) Colony morphology of strain *P. negundinis* 12020 cultured at 28°C on a PDA medium plate for 7 days. (**B**) Neighbour-joining tree of *P. negundinis* 12020 based on ITS sequences. Numbers at nodes indicate levels of bootstrap support (%) based on a neighbour-joining analysis of 1000 resampled datasets; only values >50% are shown. NCBI accession numbers are provided in parentheses.

## Fig. S2 Overview of the molecular networking generated from LC-MS/MS dataset of strain *P. negundinis* 12020.

## Fig. S3 Secondary MS fragmentation analysis of compounds 1−4 and 6−8.

## Fig. S4a HR-ESI (Pos)-MS spectrum of (±)-phaeopyridone A (1).

## Fig. S4b ^1^H NMR (600 MHz, CD_3_OD) spectrum of (±)-phaeopyridone A (**1**).

## Fig. S4c ^13^C NMR spectrum (150 MHz, CD_3_OD) of (±)-phaeopyridone A (1).

## Fig. S4d HSQC spectrum of (±)-phaeopyridone A (1).

## Fig. S4e HMBC spectrum of (±)-phaeopyridone A (1).

## Fig. S4f ^1^H−^1^H COSY spectrum of (±)-phaeopyridone A (1).

## Fig. S4g The chiral separation result of (±)-phaeopyridone A (1).

## Fig. S5a HR-ESI (Pos)-MS spectrum of (±)-phaeopyridone B (2).

## Fig. S5b ^1^H NMR spectrum (600 MHz, CD_3_OD) of (±)-phaeopyridone B (2).

## Fig. S5c ^13^C NMR spectrum (150 MHz, CD_3_OD) of (±)-phaeopyridone B (**2**).

## Fig. S5d HSQC spectrum of (±)-phaeopyridone B (2).

## Fig. S5e HMBC spectrum of (±)-phaeopyridone B (2).

## Fig. S5f ^1^H−^1^H COSY spectrum of (±)-phaeopyridone B (2).

## Fig. S5g The chiral separation result of (±)-phaeopyridone B (2).

## Fig. S6a HR-ESI (Pos)-MS spectrum of (±)-phaeopyridone C (3).

## Fig. S6b ^1^H NMR spectrum (600 MHz, CD_3_OD) of (±)-phaeopyridone C (3).

## Fig. S6c ^13^C NMR spectrum (150 MHz, CD_3_OD) of (±)-phaeopyridone C (3).

## Fig. S6d HSQC spectrum of (±)-phaeopyridone C (3).

## Fig. S6e HMBC spectrum of (±)-phaeopyridone C (3).

## Fig. S6f ^1^H−^1^H COSY spectrum of (±)-phaeopyridone C (3).

## Fig. S6g The chiral separation result of (±)-phaeopyridone C (3).

## Fig. S7a HR-ESI (Pos)-MS spectrum of berkeleyamide E (4).

## Fig. S7b ^1^H NMR (600 MHz, DMSO) spectrum of berkeleyamide E (4).

## Fig. S7c ^13^C NMR (150 MHz, DMSO) spectrum of berkeleyamide E (4).

## Fig. S7d HSQC spectrum of berkeleyamide E (4).

## Fig. S7e HMBC spectrum of berkeleyamide E (4).

## Fig. S7f ^1^H−^1^H COSY spectrum of berkeleyamide E (4).

## Fig. S7g NOESY spectrum of berkeleyamide E (4).

## Fig. S8a HR-ESI (Pos)-MS spectrum of phaeopyranone (5).

## Fig. S8b ^1^H NMR (600 MHz, CD_3_OD) spectrum of phaeopyranone (5).

## Fig. S8c ^13^C NMR (150 MHz, CD_3_OD) spectrum of phaeopyranone (5).

## Fig. S9a HR-ESI (Pos)-MS spectrum of (8*S*,9*S*)-dihydroisoflavipucine (6).

## Fig. S9b ^1^H NMR (600 MHz, CD_3_OD) spectrum of (8*S*,9*S*)-dihydroisoflavipucine (6).

## Fig. S9c ^13^C NMR (150 MHz, CD_3_OD) spectrum of (8*S*,9*S*)-dihydroisoflavipucine (6).

## Fig. S10a HR-ESI (Pos)-MS spectrum of (8*S*,9*R*)-dihydroisoflavipucine (7).

## Fig. S10b ^1^H NMR (600 MHz, CD_3_OD) spectrum of (8*S*,9*R*)-dihydroisoflavipucine (7).

## Fig. S10c ^13^C NMR (150 MHz, CD_3_OD) spectrum of (8*S*,9*R*)-dihydroisoflavipucine (7).

## Fig. S11a HR-ESI (Pos)-MS spectrum of sapinopyridione (8).

## Fig. S11b ^1^H NMR (600 MHz, CD_3_OD) spectrum of sapinopyridione (8).

## Fig. S11c ^13^C NMR (150 MHz, CD_3_OD) spectrum of sapinopyridione (8).

## Fig. S12 The reactions catalyzed by homologues of Orf12.

# Supplementary Tables

## Table S1. Enzyme function annotation of BGC of *Hypomyces roseus* 17014.

| ORF | AA length | Homolog Function (protein) | Homolog Accession | Identity (%) | Similarity  (%) |
| --- | --- | --- | --- | --- | --- |
| 1 | 286 | Short chain dehydrogenase | A0A144Y7G4.1 | 31.7 | 49.2 |
| 2 | 579 | MFS-type transporter | Q0D153.1 | 59.6 | 70.6 |
| 3 | 163 | Putative oxygenase | Q0D154.1 | 64.4 | 78.5 |
| 4 | 161 | Putative oxygenase | Q0D155.1 | 54.3 | 73.5 |
| 5 | 548 | Isoflavipucine cluster Transcription factor | Q0D158.1 | 23.9 | 36.4 |
| 6 | 438 | *α/β* hydrolase | Q4WAZ8.1 | 62.9 | 76.7 |
| 7 | 443 | Isoflavipucine cluster Transcription factor | Q0D158.1 | 49.7 | 63.1 |
| 8 | 124 | Epoxidase | A0A7L9EYL3.1 | 19.6 | 41.1 |
| 9 | 3932 | Hybrid PKS-NRPS synthetase | Q0D159.1 | 59.1 | 74.0 |
| 10 | 443 | Cytochrome P450  monooxygenase | P0DPA4.1 | 27.6 | 41.4 |
| 11 | 316 | Somaliensene A/B synthase | P0DXD5.1 | 26.4 | 41.5 |
| 12 | 609 | Dehydrogenase | A0A5C1RDA6.1 | 31.0 | 48.9 |
| 13 | 471 | FAD-linked oxidoreductase | G0R6T3.1 | 42.4 | 58.8 |
| 14 | 992 | Probable chitinase | A0A0A2J9B0.1 | 35.9 | 52.2 |
| 15 | 366 | Glycoside hydrolase | CDM30143.1 | 26.8 | 38.8 |

## Table S2. Enzyme function annotation of BGC of *P. negundinis* 12020.

| ORF | AA length | Homolog Function (protein) | Homolog Accession | Identity (%) | Similarity  (%) |
| --- | --- | --- | --- | --- | --- |
| 1 | 109 | Pyomelanin biosynthesis cluster protein | Q4WHU1.1 | 20.6 | 21.9 |
| 2 | 665 | Isoflavipucine cluster transcription factor | Q0D158.1 | 15.6 | 23.5 |
| 3 | 227 | Putative oxygenase | Q0D155.1 | 34.6 | 48.9 |
| 4 | 466 | *α/β* hydrolase psoB | Q4WAZ8.1 | 61.9 | 74.7 |
| 5 | 581 | MFS-type transporter | Q0D153.1 | 59.6 | 70.1 |
| 6 | 191 | Putative oxygenase | Q0D154.1 | 60.7 | 69.1 |
| 7 | 387 | Isoflavipucine cluster transcription factor | Q0D158.1 | 45.2 | 57.3 |
| 8 | 169 | Epoxidase | A0A7L9EYL3.1 | 31.4 | 48.0 |
| 9 | 4006 | Hybrid PKS-NRPS synthetase | Q0D159.1 | 49.6 | 65.8 |
| 10 | 769 | Het domain protein | XP_020129199.1 | 60.7 | 72.1 |
| 11 | 753 | Notoamide biosynthesis cluster protein | L7WU90.1 | 20.3 | 31.8 |
| 12 | 320 | FAD-dependent monooxygenase | KAB2573267.1 | 20.1 | 23.3 |
| 13 | 353 | Probable sugar phosphate/phosphate translocator | Q9SUV2.1 | 30.9 | 44.8 |
| 14 | 1171 | Non-canonical non-ribosomal peptide synthetase | W7N2C1.1 | 26.3 | 41.1 |
| 15 | 357 | Dehydrogenase | A0A2Z4HPZ6.1 | 45.3 | 57.8 |
| 16 | 919 | Zinc-type alcohol dehydrogenase-like protein | O94564.1 | 11.6 | 17.6 |

## Table S3. Annotation for 22 nodes in the pyridone clusters I&II from the PN12020 Featured Based Molecular Networking.

| Node (Observed  precursor *m*/*z*) | Molecular formula | Calc. *m/z* | ^a^Δ (ppm) | Δ_m/z_ from seed (8*S*,9*S*)-Dihydroisoflavipucine (**6**) | Annotation |
| --- | --- | --- | --- | --- | --- |
| 220.0966 | C_12_H_14_O_3_N | 220.0968 | 0.91 | -20.0260 | -H_4_O from **6** |
| 221.0995 | - | - | - | - | isotopic peak of 220.0966 |
| 224.0918 | C_11_H_14_O_4_N | 224.0917 | 0.45 | -16.0308 | Cpd. **1**  [M + H]^+^ |
| 224.1260 | - | - | - | - | - |
| 224.1277 | - | - | - | - | - |
| 226.1067 | C_11_H_16_O_4_N | 226.1074 | 3.10 | -14.0159 | -CH_2_ from **6** |
| 236.0521 | C_9_H_11_O_5_NNa | 236.0529 | 3.39 | -4.0705 | -C_3_H_7_ + ONa from **6** |
| 238.107 | C_12_H_16_O_4_N | 238.1074 | 1.68 | -2.0156 | Cpd. **4**  [M-H_2_O+H]^+^ |
| 238.107 | C_12_H_16_O_4_N | 238.1074 | 1.68 | -2.0156 | Cpd. **8**  [M + H]^+^ |
| 240.1223 | C_12_H_18_O_4_N | 240.1230 | 2.92 | -0.0003 | Cpd. **7**  [M + H]^+^ |
| 240.1226 | C_12_H_18_O_4_N | 240.1230 | 1.67 | - | Cpd. **6**  [M + H]^+^ |
| 241.1254 | - | - | - | - | isotopic peak of 240.1226 |
| 254.1015 | C_12_H_18_O_5_N | 254.1023 | 3.15 | 13.9789 | Cpd.**2**  [M + H]^+^ |
| 254.1016 | C_12_H_18_O_5_N | 254.1023 | 2.75 | 13.9790 | Cpd. **3**  [M + H]^+^ |
| 262.1037 | C_12_H_17_O_4_NNa | 262.1050 | 4.96 | 21.9811 | Cpd. **7**  [M + Na]^+^ |
| 262.1046 | C_12_H_17_O_4_NNa | 262.1050 | 1.53 | 21.9820 | Cpd. **6**  [M + Na]^+^ |
| 264.1205 | C_12_H_19_O_4_NNa | 264.1206 | 0.38 | 23.9979 | +HNa from **6** |
| 278.0993 | C_12_H_17_O_5_NNa | 278.0999 | 2.16 | 37.9767 | Cpd. **4**  [M + Na]^+^ |
| 479.2362 | C_24_H_35_O_8_N_2_ | 479.2388 | 5.43 | - | Cpd. **6**  [2M + H]^+^ |
| 501.2187 | C_24_H_34_O_8_N_2_Na | 501.2207 | 3.99 | - | Cpd. **6**  [2M + Na]^+^ |
| 501.2188 | C_24_H_34_O_8_N_2_Na | 501.2207 | 3.79 | - | Cpd. **7**  [2M + Na]^+^ |
| 523.2023 | C_26_H_32_O_8_N_2_Na | 523.2051 | 5.35 | - | - |

^a^Δ = (Observed *m/z* – Calc. *m/z*)/Calc. *m/z*; “-” means less possible to be clonorosein anologues.

## Table S4. Crystal data and structure refinements for (±)-phaeopyridone A−C (1−3), and phaeopyranone (5).

| Compound | **1** | **2** | **3** | **5** |
| --- | --- | --- | --- | --- |
| CCDC number | 2443614 | 2443615 | 2443616 | 2443617 |
| Empirical Formula | C_11_H_13_NO_4_ | C_12_H_15_NO_5_ | C_12_H_15_NO_5_ | C_7_H_8_O_4_ |
| Formula Weight | 223.22 | 253.25 | 253.25 | 156.13 |
| Temperature | 299 K | 295 K | 295 K | 277 K |
| Cu *K*α radiation | 1.54178 Å | 1.54178 Å | 1.54178 Å | 1.54178 Å |
| Crystal System | Triclinic | Monoclinic | Monoclinic | Monoclinic |
| Space group | *P*1¯ | *P*2_1_/c | *P*2_1_/c | *P*2_1_/c |
| *a* | 8.5265 (4) Å | 12.7118(10) | 13.0103 (9) Å | 5.1435 (2) Å |
| *b* | 11.1176 (6) Å | 9.8062 (7) | 10.2377 (7) Å | 10.1132 (3) Å |
| *c* | 12.6821 (7) Å | 10.0629 (7) | 10.0257 (6) Å | 13.8103 (5) Å |
| α | 64.020 (2) ° | 90° | 90° | 90° |
| β | 89.310 (3) ° | 93.611 (5) ° | 110.829 (4) ° | 100.489 (2)° |
| γ | 80.993 (3) ° | 90° | 90° | 90° |
| V | 1065.21 (10) Å^3^ | 1251.90 (16) Å^3^ | 1248.11 (15) Å^3^ | 706.37 (4) Å^3^ |
| Z | 4 | 4 | 4 | 4 |
| Calculated density | 1.392 mg·m^-3^ | 1.333 mg·m^-3^ | 1.348 mg·m^-3^ | 1.468 mg·m^-3^ |
| Absorption coefficient | 0.90 mm^-1^ | 0.89 mm^-1^ | 0.89 mm^-1^ | 1.05 mm^-1^ |
| F (0 0 0) | 472 | 528 | 536 | 328 |
| Crystal Size | 0.28 × 0.18 × 0.06 mm | 0.28 × 0.18 × 0.06 mm | 0.28 × 0.18 × 0.06 mm | 0.12 × 0.11 × 0.1 mm |
| *θ* Range | 4.5−65.1° | 5.7−68.5° | 3.6−63.7° | 5.5−68.3° |
| Limiting indices |  |  |  |  |
| *h* | -10−9 | -15−13 | -15−15 | -5−6 |
| *k* | -12−13 | -11−10 | -12−11 | -11−12 |
| *l* | -14−14 | -12−10 | -12−11 | -16−16 |
| Measured Reflections | 16718 | 10385 | 14572 | 11031 |
| Independent reflections | 3493 | 2253 | 2211 | 1265 |
| Data/restraints/parameters | 3493 /0/297 | 2253/1/168 | 2211 /26/ 188 | 1265/0/101 |
| Refinement on *F*^2^ | Least-squares matrix: full | Least-squares matrix: full | Least-squares matrix: full | Least-squares matrix: full |
| Goodness-of-fit on F^2^ | 1.112 | 1.149 | 1.167 | 1.096 |
| Final *R* indexes [*I*>2σ (I)] | *R*_1_ = 0.058, w*R*_2_ = 0.139 | *R*_1_ = 0.113, w*R*_2_ = 0.366 | *R*_1_ = 0.080, w*R*_2_ = 0.215 | *R*_1_ = 0.043, w*R*_2_ = 0.107 |
| Final *R* indexes [all data] | *R*_1_ = 0.063, w*R*_2_ = 0.142 | *R*_1_ = 0.123, w*R*_2_ = 0.374 | *R*_1_ = 0.120, w*R*_2_ = 0.244 | *R*_1_ = 0.048, w*R*_2_ = 0.112 |
| Largest diff. peak/hole | 0.24/-0.22 e.Å^-3^ | 0.65/-0.32 e.Å^-3^ | 0.59/-0.40 e.Å^-3^ | 0.16/-0.24 e.Å^-3^ |

## Table S5. NMR Spectroscopic Data (CD_3_OD, 600 MHz for ^1^H, 150 MHz for ^13^C) of (8*S*,9*S*)-dihydroisoflavipucine (6), (8*S*,9*R*)-dihydroisoflavipucine (7), and sapinopyridione (8).

| Pos. | **6** | | **7** | | **8** | | |
| --- | --- | --- | --- | --- | --- | --- | --- |
|  | *δ*_H_ (*J* in Hz) | *δ*_C_, type | *δ*_H_ (*J* in Hz) | *δ*_C_, type | *δ*_H_ (*J* in Hz) | | *δ*_C_, type |
| 2 |  | 157.9, C |  | 157.9, C | | 3.92, s | 69.1, CH |
| 3 |  | 132.6, C |  | 132.6, C | |  | 61.6, C |
| 4 |  | 155.0, C |  | 155.0, C | |  | 169.0, C |
| 5 | 6.11, s | 95.0, CH | 6.11, s | 95.0, CH | |  |  |
| 6 |  | 143.5, C |  | 143.5, C | |  | 159.9, C |
| 7 | 2.27, s | 18.8, CH_3_ | 2.27, s | 18.8, CH_3_ | | 5.60, s | 106.6, CH |
| 8 | 6.05, d (2.5) | 115.8, CH | 6.05, d (2.5) | 115.8, CH | |  | 188.9, C |
| 9 | 3.88, d (10.3) | 70.5, CH | 3.88, d (10.3) | 70.5, CH | |  | 208.0, C |
| 10 | 1.56 (ddd,14.4,10.9,4.4) | 40.4, CH_2_ | 1.56 (ddd,14.4,10.9,4.4) | 40.4, CH_2_ | | 3.24, m | 45.6, CH |
|  | 1.34 (ddd,13.4,9.6,2.8) |  | 1.34 (ddd,13.4,9.6,2.8) |  |  |  |  |
| 11 | 1.89, m | 25.2, CH | 1.89, m | 25.2, CH | | 1.79, m | 26.4, CH_2_ |
|  |  |  |  |  |  | 1.48, dq (15.2, 7.4) |  |
| 12 | 0.94, d (6.5) | 21.8, CH_3_ | 0.94, d (6.5) | 21.8, CH_3_ | | 0.98, t (7.4) | 11.5, CH_3_ |
| 13 | 0.98, d (6.6) | 24.0, CH_3_ | 0.98, d (6.6) | 24.0, CH_3_ | | 1.03, d (6.4) | 13.1, CH_3_ |
| 14 |  |  |  |  | | 2.19, s | 20.5, CH_3_ |

**Table S6.** DP4 probability of ^13^C NMR chemical shifts of **4a** (5*R**,8*R**,9*R**,10*S**) and **4b** (5*R**,8*R**,9*R**,10*R**).

| No. | *δ*exp | *δ*cal | | *δ*scal | | Corrected error (Δ*δ*) | | t distribution | | Probability | |
| --- | --- | --- | --- | --- | --- | --- | --- | --- | --- | --- | --- |
|  |  | **4a** | **4b** | **4a** | **4b** | **4a** | **4b** | **4a** | **4b** | **4a** | **4b** |
| 1 | 16.7 | 17.76 | 17.6 | 15.7 | 15.0 | 1.0 | 1.7 | 0.66 | 0.76 | 0.34 | 0.24 |
| 2 | 195.0, | 202.51 | 200.0 | 195.5 | 193.0 | -0.5 | 2.0 | 0.58 | 0.80 | 0.42 | 0.20 |
| 3 | 103.9 | 108.69 | 107.3 | 104.2 | 102.6 | -0.3 | 1.3 | 0.55 | 0.71 | 0.45 | 0.29 |
| 4 | 200.2 | 206.23 | 203.7 | 199.1 | 196.6 | 1.1 | 3.6 | 0.55 | 0.71 | 0.32 | 0.07 |
| 5 | 96.3 | 103.6 | 102.4 | 99.2 | 97.7 | -2.9 | -1.4 | 0.88 | 0.73 | 0.12 | 0.27 |
| 6 | 164.5 | 168.53 | 166.3 | 162.4 | 160.1 | 2.1 | 4.4 | 0.81 | 0.96 | 0.19 | 0.04 |
| 7 | 87.3 | 91.53 | 90.3 | 87.5 | 85.9 | -0.2 | 1.4 | 0.53 | 0.72 | 0.47 | 0.28 |
| 8 | 71.3 | 76.95 | 77.7 | 73.3 | 73.6 | -2.0 | -2.3 | 0.80 | 0.83 | 0.20 | 0.17 |
| 9 | 41.1 | 42.62 | 46.3 | 39.9 | 43.0 | 1.2 | -1.9 | 0.70 | 0.79 | 0.30 | 0.21 |
| 10 | 23.3 | 28.9 | 26.4 | 26.5 | 23.6 | -3.2 | -0.3 | 0.91 | 0.55 | 0.09 | 0.45 |
| 11 | 12.4 | 13.45 | 13.6 | 10.5 | 10.1 | 1.9 | 2.3 | 0.79 | 0.83 | 0.21 | 0.17 |
| 12 | 13.4 | 17.76 | 17.6 | 11.5 | 11.1 | 1.8 | 2.2 | 0.77 | 0.82 | 0.23 | 0.18 |
| Product of probabilities | | | | | | | | | | 6.01E-08 | 1.59E-09 |
| Bayes’s theorem probability (%) | | | | | | | | | | 97.43% | 2.57% |

## Table S7. Antimicrobial activity of compounds 1−7.

| **Compounds** | **MIC (μg/mL)** | | | | | | |  |
| --- | --- | --- | --- | --- | --- | --- | --- | --- |
|  | ***C. albicans*** | ***E. coli*** | ***S. aureus*** | ***E*. *tarda*** | ***B*. *cereus*** | ***A*. *baumannii*** | ***E. aerogenes*** | ***M. tuberculosis*** |
| **1** | >100 | >100 | >100 | >100 | >100 | >100 | >100 | >100 |
| **2** | >100 | >100 | >100 | >100 | >100 | >100 | >100 | >100 |
| **3** | >100 | >100 | >100 | >100 | >100 | >100 | >100 | >100 |
| **4** | NT | NT | NT | >100 | >100 | >100 | >100 | NT |
| **5** | NT | NT | NT | >100 | >100 | >100 | >100 | 6.25 |
| **6** | >100 | >100 | >100 | >100 | >100 | >100 | >100 | NT |
| **7** | >100 | >100 | >100 | >100 | >100 | >100 | >100 | NT |
| Positive control | 1^a^ | 0.6^b^ | 1.2^c^ | 0.6^b^ | 3.1^b^ | 0.6^b^ | 0.6^b^ | 0.03^d^ |

^a^Fluconazole, ^b^Ciprofloxacin, ^c^Vancomycins, ^d^Isonicotinylhydrazide, NT: no test

## Table S8. The sequences selected for the phylogenetic analysis.

| **Sequence Name** | **Sequence ID** |
| --- | --- |
| Fsr3 | S0DQN6.1 |
| MdpD | Q5BH33.1 |
| DmxR9 | A0A4P8DJF6.1 |
| NsrK | A0A2I1C3T9.1 |
| CPUR_05423 | M1W850.1 |
| AacuC | A0A1L9WLG2.1 |
| PboD | A0A0C1C427.1 |
| NotI | E1ACQ4.1 |
| NotI′ | L7WR46.1 |
| FmqB | Q4WLW7.1 |
| NodY2 | A0A2I6PIZ8.1 |
| NodY1 | A0A2I6PJ01.1 |
| AspB | P0DOW1.1 |
| OpaC | A0A0C1BV72.1 |
| NotB | E1ACP7.2 |
| NotB′ | L7WME6.1 |
| NanF | A0A6G9KH61.1 |
| phomE | A0A8K1Y6D8.1 |
| phomE′ | A0A142I725.1 |
| OpS4 | J4VWM7.1 |
